# Disruption of thrombospondin 1/2-integrin β1 axis impairs cell adhesion and tumor growth in intrahepatic cholangiocarcinoma

**DOI:** 10.1038/s41420-026-03164-1

**Published:** 2026-06-01

**Authors:** Veronica Porreca, Ludovica Giancola, Luca Sallustio, Alessia Conigliaro, Pietro Angelone, Roberta Marrone, Francesco Greco, Dionino Marco Giangrande, Giulio Bontempi, Michele Stella, Biagio Palmisano, Giuseppina Mignogna, Martina Meucci, Fabio Melandro, Gianluca Mennini, Massimo Rossi, Mara Riminucci, Alessandro Corsi, Helena Stabile, Marco Ragusa, Antonio Filippini, Valerio Fulci, Raffaele Strippoli, Bruno Maras, Carmine Mancone

**Affiliations:** 1 Department of Molecular Medicine, Sapienza University of Rome, Viale del Policlinico 155, 00161 Rome, Italy; 2 National Institute for Infectious Diseases L. Spallanzani, IRCCS, Via Portuense, 292, 00149 Rome, Italy; 3 Department of Biomedical and Biotechnological Sciences, Section of Biology and Genetics “G. Sichel”, University of Catania, 95123 Catania, Italy; 4 Department of Biochemistry Science, Sapienza University of Rome, P.le Aldo Moro 5, 00185 Rome, Italy; 5 Department of Anatomy, Histology, Forensic Medicine and Orthopedics, Unit of Histology and Medical Embryology, Sapienza University, Rome, Italy; 6 General Surgery and Organ Transplantation Unit, Department of General Surgery and Surgical Specialties P. Stefanini, Sapienza University of Rome, Rome, Italy

**Keywords:** Bile duct cancer, Integrins

## Abstract

Elevated expression of THBS1 and THBS2 in intrahepatic cholangiocarcinoma (iCCA) contributes to tumor growth and metastatic dissemination. Both proteins are predominantly produced by cancer-associated fibroblasts (CAFs) and iCCA cells, enhancing the interaction of malignant cholangiocytes with the extracellular matrix (ECM). Here, we identify integrin α3β1 and α6β1 as the cognate receptors for THBS1 and THBS2 on iCCA cell surface. Disruption of the THBS1-integrin β1 axis via monoclonal antibodies, THBS1-derived peptides, or THBS1 knockout (KO) iCCA cells reduces autocrine and paracrine integrin β1 activation, resulting in decreased ECM adhesion in both two-dimensional and three-dimensional assays. Loss of endogenous THBS1 also alters cell morphology, weakens intracellular junctions, and prevents tumor formation in mouse xenograft models. These findings identify the THBS1/THBS2-integrin β1 axis as a key driver of iCCA cell adhesion and malignancy, supporting its potential as a therapeutic target for iCCA-specific interventions.

## Introduction

Cell adhesion to the extracellular matrix (ECM) is essential for cancer cell survival, proliferation, and motility [1]. To strengthen their interaction with the ECM, cancer cells remodel their plasma membrane to express adhesion molecules with higher binding affinity than those of normal cells [2]. The increased adhesiveness is further reinforced by matricellular proteins (MCPs) such as thrombospondins (THBSs), osteonectin (SPARC), periostin, CCN proteins, and osteopontin. These proteins are abundantly released within the tumor microenvironment (TME), where they bridge cancer cells to the major constituents of the ECM [3, 4]. The interplay between the adhesion molecules and MCPs is critical for regulating cancer cell anoikis and driving local invasion and metastasis [2, 5, 6]. Thus, understanding the adhesion molecules expressed on the surface of cancer cells and their interaction with the release of MCPs within the TME is increasingly recognized as crucial for counteracting cancer progression and improving patient outcomes through targeted therapies.

Thrombospondin 1 (THBS1) and 2 (THBS2) are multifunctional MCPs that mediate cell-cell and cell-ECM communications in numerous cancer cell types [7]. Recently, THBS1 and THBS2 have emerged as two crucial drivers in intrahepatic cholangiocarcinoma (iCCA) progression [8–10]. iCCA is a highly lethal malignancy that exerts its aggressiveness through the high proliferating and invasive abilities of the malignant cholangiocytes [11]. These abilities drive silent and rapid intrahepatic expansion and metastasis in lymph nodes, often precluding curative treatment options [12]. In iCCA, THBS1 and THBS2 are overexpressed by both the cancer-associated fibroblasts (CAFs) and iCCA cells, and THBS1 overexpression correlates with poor prognosis in iCCA [8, 10, 13]. Both the THBSs contribute to the trans-differentiation of vascular endothelial cells into a lymphatic-like phenotype, thereby suppressing angiogenesis while promoting iCCA-associated neo-lymphangiogenesis [8]. Most importantly, THBS1 and THBS2 directly promote iCCA cell adhesion and, consequently, influence the iCCA cell malignant behavior [9].

THBS1 and THBS2 are homotrimeric multidomain proteins sharing 62% sequence identity in human [14]. Each subunit is a multidomain chain where the N- and C-terminal globular regions are interconnected by a central stalk composed of the procollagen domain, three type 1 repeats (properdin-like), three type II (EGF-like), and seven type 3 repeats (calcium-binding) [14]. They can bridge multiple cell surface receptors, including CD36, CD47, integrin β1 and β3, certain syndecans, LDL receptor-related protein-1 (LRP1), to ECM proteins such as collagens, fibronectin, and laminins [14]. In solid tumors, integrin β1 and β3, CD36, and CD47 have emerged as key mediators in THBSs-driven cancer cell adhesion [15]. In many solid cancers, THBS1 and THBS2 promote tumor cell adhesion through interactions with integrin α3β1, α4β1, and αvβ3 [16–22]. Beyond integrins, THBSs engage CD36 receptor to promote cell adhesion in cutaneous squamous cell carcinoma and in pancreatic ductal adenocarcinoma [22] while the THBS2-CD47 axis drives cell adhesion and progression in colorectal cancer [23]. Although these adhesion molecules have well-characterized receptor partners in other tumor types, the specific receptors mediating THBS1 and THBS2-driven adhesion in iCCA remain to be elucidated.

Here, we identify integrin α3β1 and α6β1 as the cell surface receptors engaged by THBS1 and THBS2 in iCCA. We show that autocrine and exogenous THBS1 and THBS2 mediate the outside-in activation of these integrins β1 in iCCA cell lines. THBS1 knockout (KO) iCCA cells generated using CRISPR/Cas9 approach markedly reduced integrin β1 activation and, consequently, decreased adhesion to the ECM. Loss of THBS1 also altered cell morphology by reprogramming the expression of a core set of adhesive and cytoskeletal genes. In in vitro three-dimensional (3D) cell cultures, the THBS1 KO negatively impacts tumor spheroid formation, while in xenograft models, THBS1 KO iCCA cells fail to generate tumor masses, underscoring the essential role of THBS1 in tumor growth. Finally, antagonist THBS1-derived peptides disrupt the interaction of THBS1 and THBS2 to integrin α3β1 and α6β1, strongly reducing the iCCA cell adhesion in 2D and 3D systems. Together, these findings reveal a crucial role for THBS1 and THBS2 in the iCCA cell adhesiveness and highlight a promising therapeutic strategy to attenuate iCCA progression.

## Results

### Integrin β1 activation is required for the iCCA cell adhesion

**Fig. 1:**
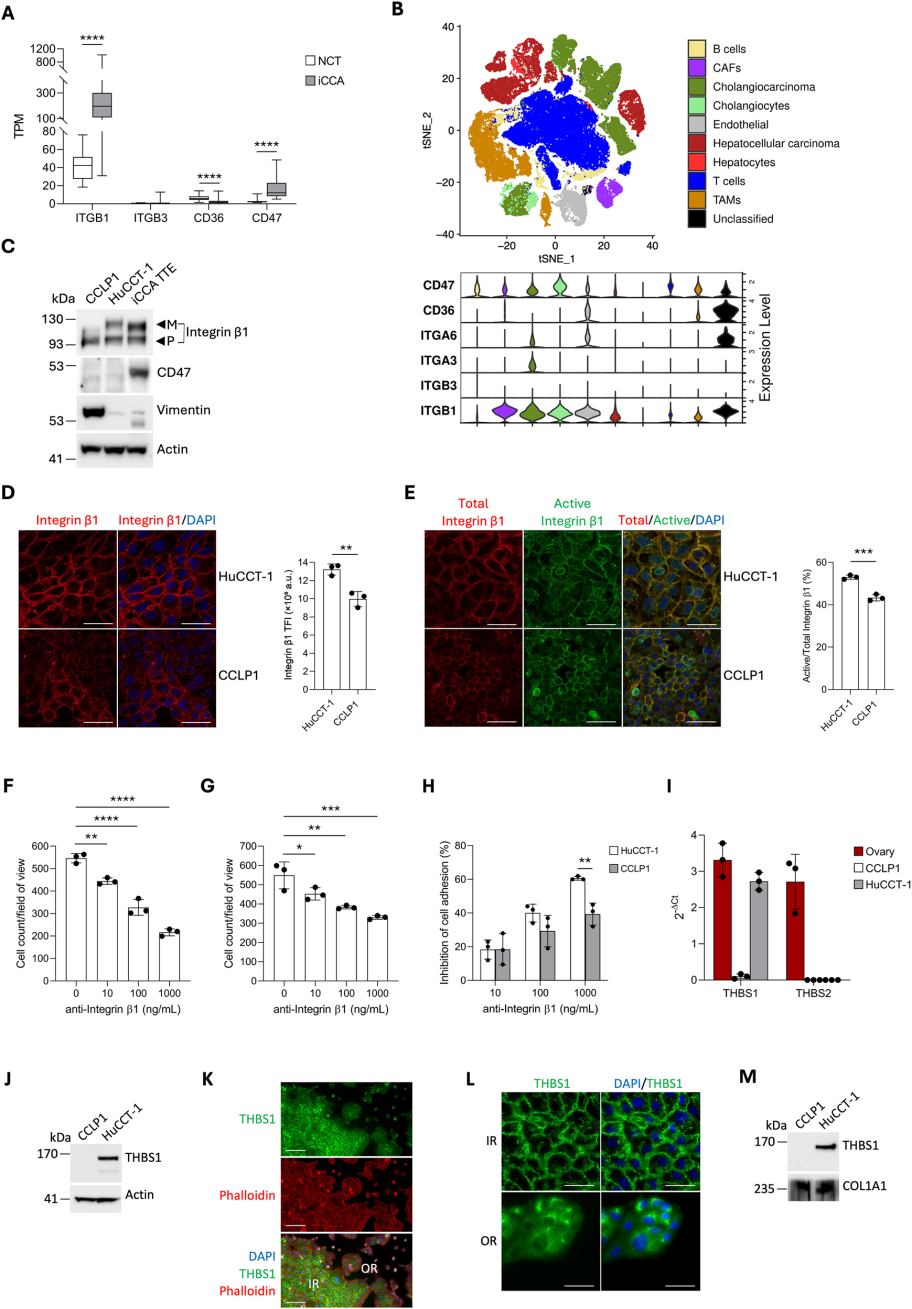
Activated integrin β1 mediates iCCA cell adhesion. **A** Bulk RNA-seq data from the GEO dataset GSE107943 were retrieved and analyzed to assess the expression of ITGB1, ITGB3, CD36, and CD47 between iCCA and NCT tissues from iCCA patients. Box plots show transcripts per million (TPM) values. **B** Single-cell RNA-seq data from publicly available GEO datasets GSE151530 and GSE138709 derived from iCCA and HCC patients were analyzed. t-SNE plot shows clustering of individual cells according to tSNE_1 (x-axis) and tSNE_2 (y-axis), with clusters color-coded by cell type or expression profile. **C** Immunoblot analysis of integrin β1, CD47, and vimentin in the CCLP1 and HuCCT-1 cell lines. iCCA TTE and actin were used as positive and loading controls, respectively. Arrowheads indicate integrin β1 mature (M) and precursor (P) forms. **D** Illustrative images of HuCCT-1 and CCLP1 immunostained for integrin β1 with DAPI-labeled nuclei. Total fluorescence intensity (TFI; arbitrary units, a.u.) was quantified as the sum of all pixel intensities across regions of interest (ROIs). **E** Representative confocal images of total and active integrin β1 in both iCCA cell lines. The fraction of active integrin β1 relative to the total signal is reported as a percentage. Scale bars, 50 μm. Adhesion assay of HuCCT-1 (**F**) and CCLP1 (**G**) cells upon treatment with anti-integrin β1 blocking antibody at the indicated concentrations. **H** Percentage of cell adhesion inhibition in HuCCT-1 and CCLP1 cells following treatments with anti-integrin β1 blocking antibody at the indicated concentrations. **I** THBS1 and THBS2 transcript abundance in the indicated iCCA cell lines quantified by qRT-PCR. Commercial human ovary RNA was used as a positive control. Bar plots show 2^−ΔCt^ values normalized to GAPDH. **J** Immunoblot detection of THBS1 in total cell lysates obtained from HuCCT-1 and CCLP1 cells. Actin was used as a loading control. **K** Representative IF staining of HuCCT-1 cells with phalloidin, THBS1, and DAPI-labeled nuclei. The inner region (IR) and outer region (OR) are indicated. Scale bar, 100 µm. Phalloidin was used to highlight cell shape. **L** Representative IF staining for THBS1 in HuCCT-1 cells at the OR and IR of the cell layer. Scale bar, 50 µm. **M** Immunoblot detection of THBS1 in conditioned medium obtained from HuCCT-1 and CCLP1 cells. COL1A1 was used as loading control. All data are presented as mean ± SD. Unless otherwise specified, quantifications derive from three biologically independent experiments. Statistical significance was assessed using two-tailed Student’s t test for pairwise comparisons (**A**, **D**, **E**, **H**) and one-way ANOVA with Dunnett’s post-hoc test for cell adhesion assays (**F**, **G**). *p < 0.05; **p < 0.01; ***p < 0.001; **** p < 0.0001.

Thrombospondins (THBSs) are known to regulate cancer cell adhesion through key receptors, including integrin β1, integrin β3, CD36, and CD47. To investigate their potential involvement in iCCA, we first analyzed RNA expression levels of these molecules in silico by retrieving expression data from 30 tumoral and matched surrounding non-cancerous (NCT) tissues of patients with iCCA [24]. As shown in Fig. 1A, high transcriptional levels of integrin β1 were observed in tumoral tissues compared to NCT. CD47 mRNA levels were also upregulated in iCCA samples. Conversely, integrin β3 and CD36 showed lower expression levels and were downregulated in iCCA samples. By WB analysis of total tissue extracts (TTE) of iCCA patients, we confirmed that integrin β1 was the most expressed protein among the receptors examined in iCCA specimens (Supplementary Fig. 1A). By in silico analysis of single-cell RNA datasets derived from iCCA and hepatocellular carcinoma (HCC) patients [25, 26], we found that, among the putative THBSs receptors, integrin β1 and CD47 mRNAs were expressed in the iCCA cells population (Fig. 1B). Notably, integrin β1 resulted slightly increased in iCCA cells compared to non-malignant cholangiocytes, whereas CD47 showed the opposite trend (Fig. 1B). We subsequently analyzed the protein expression levels of the integrin β1 and CD47 on HuCCT-1 and CCLP1 cells, two established iCCA cell models that exhibit an epithelial and a mesenchymal-like phenotype, respectively (Fig. 1C). No protein expressions were observed for CD47, suggesting that sporadic CD47 expression in iCCA TTE may be related to non-cancerous cells (Fig. 1C and Supplementary Fig. 1A). On the contrary, integrin β1 was detected in both cell lines as precursor (P) and mature (M) chains, although CCLP1 showed reduced levels of the M form. Immunofluorescence (IF) staining showed a dotted pattern, typical of focal adhesions (FAs) clustering, in both iCCA cell lines, although we measured increased integrin β1 clusters in HuCCT-1 cells (Fig. 1D). Integrins exist in different conformational states that determine the receptor affinity for ECM proteins. The “bent closed” integrin represents the inactive form, with low affinity for ECM ligands, whereas a “fully extended open” integrin is active in FAs and capable of eliciting downstream signaling and cellular responses following ligand engagement [27]. Based on these observations, we hypothesized that HuCCT-1 cells express more integrin β1 active form than CCLP1. To test this hypothesis, we used an antibody specific for the activated conformation of integrin β1 [28]. IF staining showed that the HuCCT-1 cells had significantly percentage increase of active/total integrin β1 compared to CCLP1 (Fig. 1E). Both iCCA cell lines were subjected to an adhesion assay in the presence of increasing concentrations of anti-integrin β1 blocking antibodies. As expected, we observed a statistically significant decrease in the attachment of both cell lines (Fig. 1F-G). Notably, the blocking antibody were more effective on HuCCT-1 cells when compared to CCLP1, indicating that the HuCCT-1 relies more heavily on activated integrin β1, whereas CCLP1 show a lower dependence on integrin β1 for adhesion (Fig. 1H). Since integrin β1 can be engaged and activated by both THBS1 and THBS2 [14], we next asked whether the increased integrin β1 activation in HuCCT-1 cells could be correlated with THBS1 and THBS2 expression in these cells. Consistent with THBSs expression in iCCA tissues [8], THBS1 mRNA and protein expressions were observed exclusively in HuCCT-1 cells, while neither type of cells expressed THBS2 (Fig. 1I–J). Interestingly, HuCCT-1 cells showed different patterns of THBS1 expression and subcellular localization (Fig. 1K). The overall signal intensity of THBS1 was found markedly increased in the inner region (IR) of the cell layer with the respect to the outer region (OR) (Fig. 1K). Furthermore, cells at the OR displayed a cytoplasmic speckled pattern, while in the IR THBS1 was found to be distributed as a peripheral membrane protein (Fig. 1L and Supplementary Fig. 1B). Most importantly, THBS1 was released in the extracellular space, suggesting an autocrine behavior that may be responsible for the increased integrin β1 activation in these cells (Fig. 1M).

### THBS1 KO in HuCCT-1 cells negatively affects integrin β1 activation, cell adhesion and morphology

**Fig. 2:**
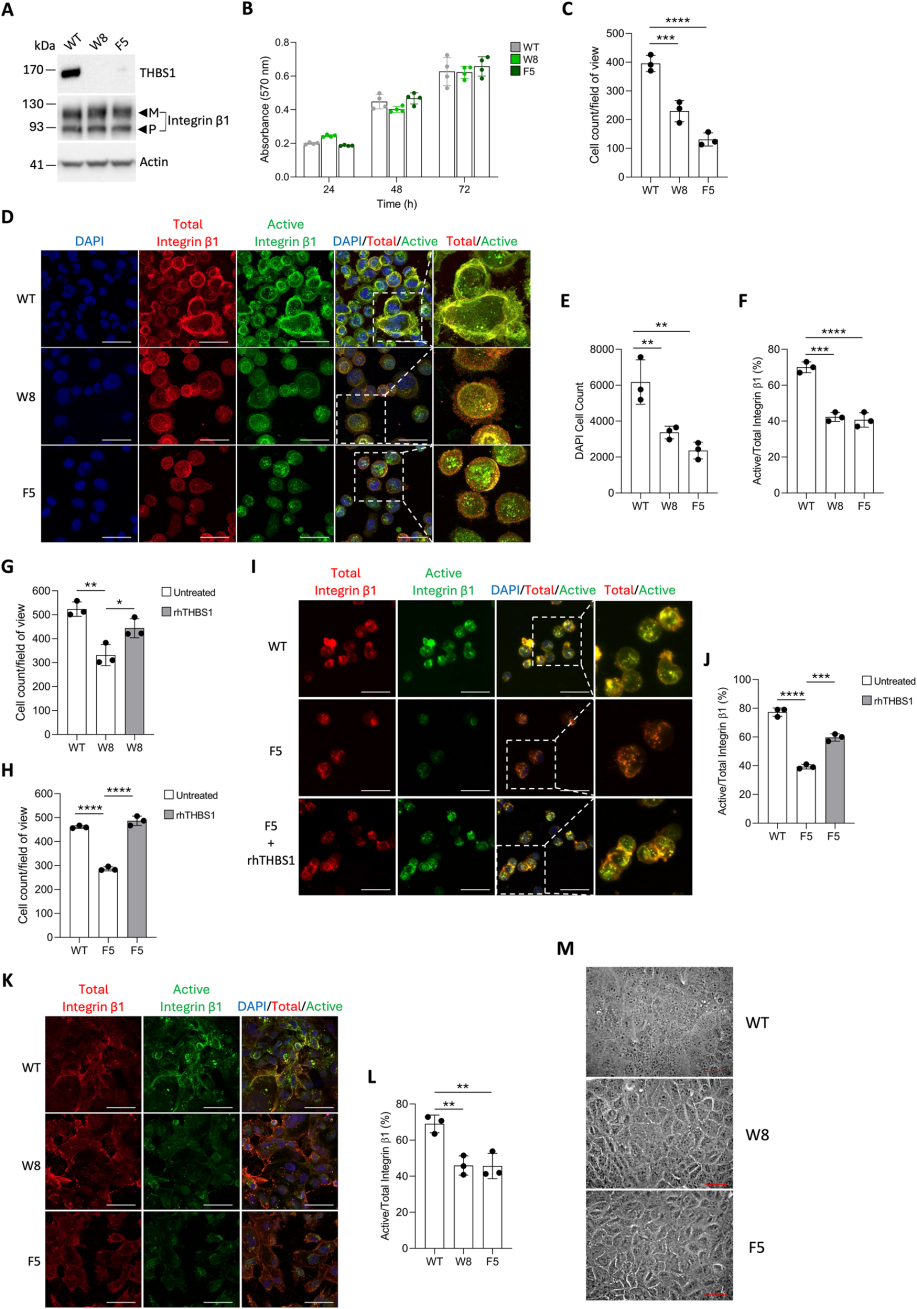
THBS1 loss impairs integrin β1 activation, ECM-cell adhesion, and monolayer integrity. **A** Representative immunoblots of THBS1 and integrin β1 in the indicated cells. Actin was used as loading control. **B** MTT cell viability assay of WT and THBS1 KO cells at various time points; each dot represents an independent measurement from four experiments. **C** Cell adhesion assay of WT, W8, and F5 cells. **D–F** Confocal analysis of integrin β1 activation in a cell adhesion assay for the indicated cells. Illustrative images of total and active integrin β1; insets highlight differences in integrin β1 activation (**D**). DAPI-stained nuclei counted (**E**). Quantitative analysis of active integrin β1 levels relative to total integrin β1 signal (**F**). Cell adhesion assay of W8 (**G**) and F5 (**H**) cells in response to 1000 ng/mL of rhTHBS1. WT cells were used as adhesiveness control. **I–J** Confocal analysis of integrin β1 activation in a cell adhesion assay of F5 cells in response to 1000 ng/mL of rhTHBS1. WT cells were used as a control of integrin β1 activation. Representative images of total and active integrin β1; insets highlight differences in integrin β1 activation (**I**). Quantitative analysis of active integrin β1 levels relative to total integrin β1 signal (**J**). **K**, **L** Confocal analysis of integrin β1 activation 24 h post-seeding in WT and THBS1 KO cells. Representative confocal images of total and active integrin β1 (**K**). Quantitative analysis of active integrin β1 levels relative to total integrin β1 signal (**L**). **M** Brightfield images of WT and THBS1 KO cells monolayers. Scale bars, 50 μm. Horizontal bars indicate mean ± SD. Unless noted, quantifications derive from three biologically independent experiments. *p* values were calculated using one-way ANOVA with Dunnett’s test for comparisons to WT (**C**, **E**, **F**, **L**) and Tukey’s test for multiple comparisons (**G**, **H**, **J**). *p < 0.05; **p < 0.01; ***p < 0.001; **** p < 0.0001.

Given our hypothesis that increased integrin β1 activation correlates with THBS1 expression and release, we reasoned that deletion of the THBS1 gene might reduce the activated conformation of integrin β1, supporting the existence of an autocrine THBS1 signaling in these cells. To this aim, we employed the CRISPR/Cas9 approach to completely KO the THBS1 gene in HuCCT-1 cells. This approach enabled the selection of two THBS1 KO subclones in CRISPR/Cas9 engineered HuCCT-1 cell line: W8 and F5. THBS1 depletion did not affect integrin β1 protein expression (Fig. 2A). We assessed the viability and the cellular adhesive properties of HuCCT-1 wild type (WT), W8 and F5 cells. THBS1 depletion did not affect the cell viability but markedly reduced cell adhesion (Fig. 2B, C). We then quantified the integrin β1 activation levels to correlate the active form with the cell adhesion data (Fig. 2D–F). After 45 min post seeding, in both THBS1 KO subclones, we observed reduced levels of activated integrin β1 associated with the decrease in cell adhesion. To confirm the role of the THBS1 autocrine signaling in the HuCCT-1 cell adhesion and integrin β1 activation, we subjected both the THBS1 KO subclones to recombinant human THBS1 (rhTHBS1) by a cellular adhesion assay. Exogenously added rhTHBS1 restored cell adhesiveness both in W8 and F5 subclones (Fig. 2G, H). Similarly, treatment with exogenous rTHBS1 increased integrin β1 activation both in F5 (Fig. 2I–J) and W8 subclones (Supplementary Fig. 2). Subsequently, we asked whether W8 and F5 cells required more time for activating integrin β1 to establish FAs. 24 h after seeding, both the THBS1 KO subclones still exhibited defective integrin β1 activation with respect to WT cells (Fig. 2K, L). Most importantly, phase contrast images of confluent WT cells show a tight, densely packed monolayer. In contrast, although THBS1 KO cells still formed a cell layer, it was less compact, with cells appearing more separated from each other, resulting in brighter cell borders (Fig. 2M).

### Loss of THBS1 expression affects actin filament and cell junction organization

**Fig. 3:**
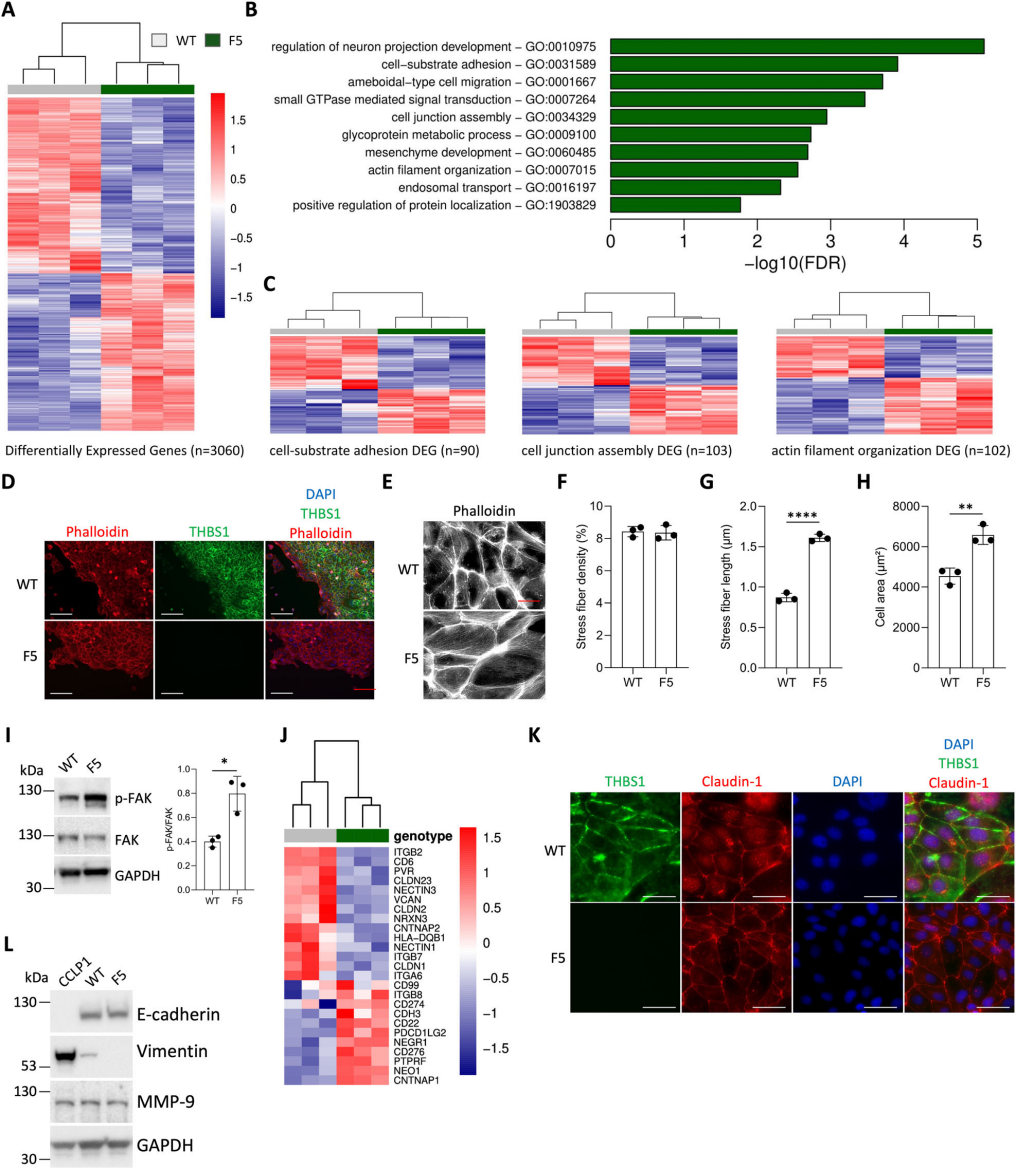
THBS1 expression influences actin filaments organization and cell-cell adhesion. **A** Heat map of 3060 DEGs identified by RNA sequencing in F5 versus WT cells (adjusted *p* < 0.05). Red and blue indicate up- and downregulated genes, respectively. **B** Top 10 significantly enriched GO biological process terms among DEGs, ranked by −log10 false discovery rate (FDR). **C** Heat maps showing expression patterns of genes associated with selected enriched GO terms related to cytoskeletal organization and cell adhesion, including cell–substrate adhesion, cell junction assembly, and actin filament organization. **D** Representative IF staining of WT and F5 cells with phalloidin, THBS1, and DAPI-labeled nuclei. Scale bar, 100 µm. **E–H** Morphometric analysis of actin stress fibers and cell area. Representative phalloidin staining is shown in grayscale to visualize actin stress fibers. Scale bar, 10 µm. **E** Actin stress fiber density was quantified as the percentage of pixels corresponding to stress fibers after image processing (**F**). Analysis of actin stress fiber length (µm) (**G**) and cell area (µm²) (**H**). **I** Representative immunoblot analysis of total and phosphorylated FAK in the indicated cells; quantification reports the ratio of p-FAK to total FAK. GAPDH was used as a loading control. **J** Heatmap depicting DEGs belonging to the “KEGG_CELL_ADHESION_MOLECULES_CAMS” GSEA gene set. **K** Representative IF staining of WT and F5 cells with THBS1, claudin-1, and DAPI-labeled nuclei. Scale bar, 50 µm. **L** Immunoblot analysis of E-cadherin, vimentin, and MMP-9 in WT and F5 cells. CCLP1 cell line and GAPDH served as positive and loading controls, respectively. Bar graphs represent mean ± SD from three independent experiments. Statistical significance was assessed using a two-tailed Student’s t test for pairwise comparisons. *p < 0.05; **p < 0.01; **** p < 0.0001.

To gain insight into the THBS1 KO-induced molecular and morphological changes, we performed RNA sequencing (RNA-seq) on WT cells and on one of the two THBS1 KO subclones (F5) used as a representative KO clone. Principal Component Analysis of RNA-seq data revealed that the major source of variance in the dataset, as expected, is associated with the genotypic difference (Supplementary Fig. 3A). This is corroborated by the sample correlation matrix (Supplementary Fig. 3B), which clearly showed that gene expression is strongly similar in the F5 biological replicates. Testing for differential gene expression between F5s and WTs, we obtained the identification of 3060 Differentially Expressed Genes (DEGs) using an adjusted p-value threshold of 0.05 (Fig. 3A, Supplementary Fig. 3A, and Supplementary Table 1). The 3060 DEGs were functionally annotated against Gene Ontology Biological Process (GO BP) categories, identifying 51 enriched GO BP categories (Supplementary Table 2) using a False Discovery Rate (FDR) threshold of 0.05. Redundant GO BP categories were removed using the “weighted set cover” algorithm implemented in WebGestalt webserver [29], thus resulting in 20 non-redundant GO BP categories significantly enriched (Supplementary Table 3). The top 10 enriched categories are reported in Fig. 3B. These included “cell-substrate adhesion”, “cell junction assembly” and “actin filament organization”, pinpointing a severe effect of THBS1 loss on the tightly interconnected pathways regulating adhesion and actin cytoskeleton (Fig. 3C).

We first gained insights into the effects of THBS1 expression on actin filament organization (Fig. 3D, E). No differences in actin stress fiber density were observed between WT and F5 cells (Fig. 3F). However, F5 cells showed longer actin stress fibers and increased cellular area when compared to WT cells (Fig. 3G, H). Actin stress fiber remodeling is known to be associated with reduced FA contacts, whose turnover leads to focal adhesion kinase (FAK) autophosphorylation [30, 31]. Consistent with this framework, we detected increased levels of phospho-FAK (p-FAK) (Fig. 3I), which, however, did not influence either cell migration or integrin β1 internalization (Supplementary Fig. 3D-E). Moreover, increased p-FAK levels were observed also in adherent HuCCT-1 cells subjected to anti-integrin β1 blocking antibody (Supplementary Fig. 3F). On the contrary, FAK autophosphorylation was downregulated in HuCCT-1 cells transfected with siRNA targeting integrin β1 (Supplementary Fig. 3G), indicating that the loss of integrin β1 prevents focal adhesion complex formation and, consequently, FAK recruitment.

We then investigated the THBS1 KO-induced dysregulation of cell junction assembly. Specifically, we focused on cell adhesion molecules (CAMs) involved in cell-cell contacts. RNAseq data highlighted the downregulation of transcripts encoding for protein components of tight (i.e.: claudin-1, -2, -23) and adherens (nectin-1 and -3) junctions (Fig. 3J) [32, 33]. Since claudin-1 expression and nuclear localization are associated with cellular transformation and metastatic behavior in many cancers [34–38], we gained insights into the claudin-1 expression in WT and F5 cells. In WT, in addition to the membrane-associated signal, claudin-1 was predominantly localized into the nucleus, suggesting a potential regulatory role (Fig. 3K). In THBS1 KO cells, claudin-1 was mainly localized at the plasma membrane and the cells appeared more scattered with reduced adhesion to the ECM. These morphological changes may suggest that these cells with reduced adhesion are undergoing epithelial-to-mesenchymal transition (EMT). To investigate this possibility, we analyzed the expressions of EMT markers and matrix metalloproteinases 9 (MMP-9). In F5 cells, a strong downregulation of vimentin was detected, while no changes in E-cadherin and MMP-9 expression were observed (Fig. 3L and Supplementary Fig. 3C). These findings indicate that loss of THBS1 expression does not induce EMT and the observed scattering likely reflects altered cell-ECM adhesion and cytoskeletal remodeling rather than increased malignancy.

### THBS1 KO iCCA cells fail to generate tumor masses in xenograft mouse models

**Fig. 4:**
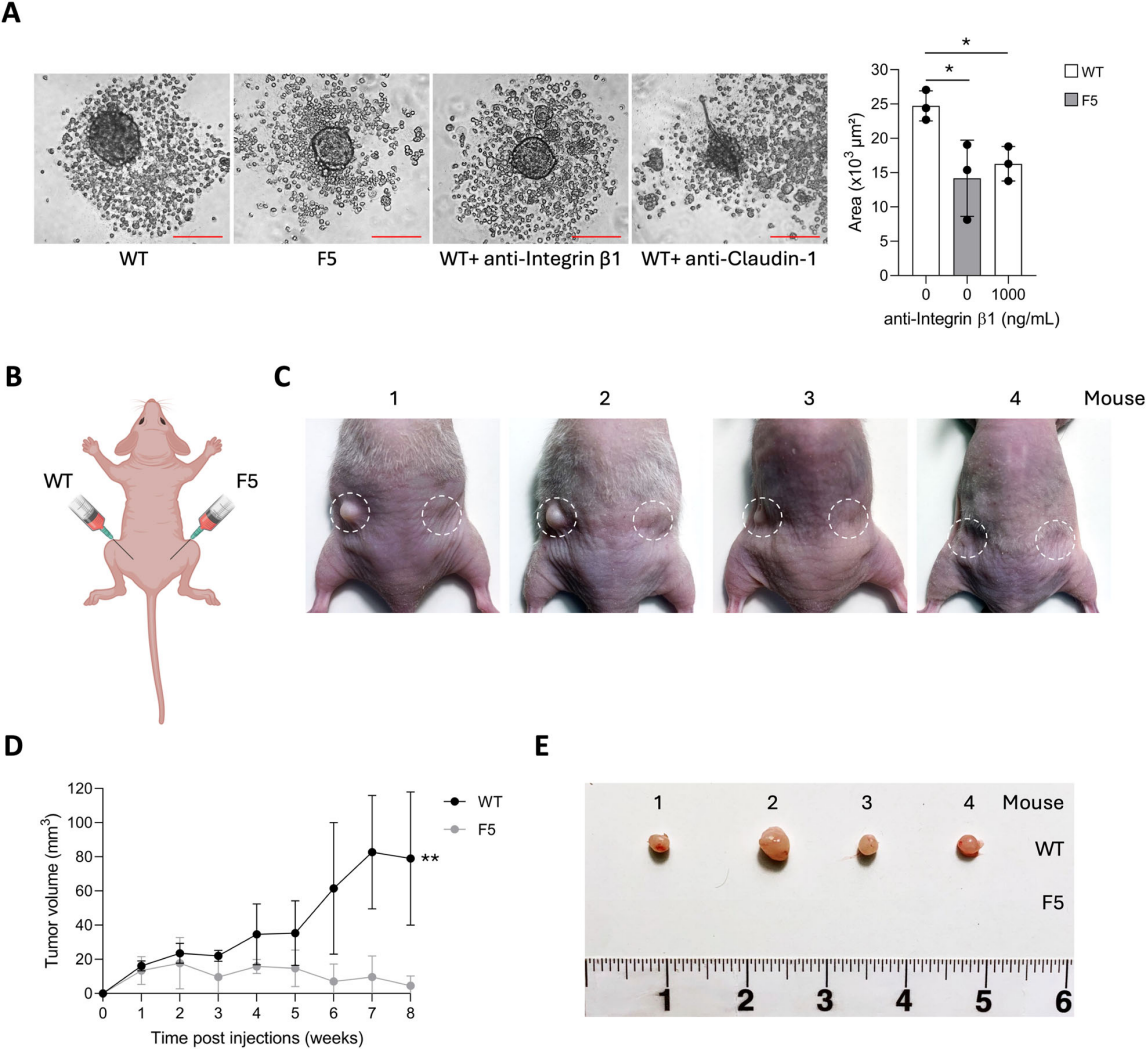
THBS1 deficiency impairs spheroid formation and tumor growth in a mouse xenograft model. **A** Representative brightfield images of spheroids generated from WT, F5, and WT cells treated with either anti-integrin β1 blocking or anti-integrin claudin-1 antibodies (1000 ng/mL). The bar graph shows mean ± SD spheroid area (µm²) measured 7 days after seeding. Scale bars = 200 µm. Statistical significance was assessed by one-way ANOVA with Tukey’s multiple comparisons test. *p < 0.05. **B** Schematic illustration of the bilateral inoculation of WT or F5 cells in the xenograft mouse model. Created with BioRender.com with granted permission and license. **C** Representative images of mice 8 weeks following cell injection. **D** Tumor volumes (mm^3^) were measured weekly before recovery. Data are presented as mean ± SD. Statistical significance was assessed using a two-tailed Student’s t-test for pairwise comparisons. **E** Illustrative images of xenograft tumors recovered from the right and left flanks of each mouse after 8 weeks.

We next investigated the effects of the reduced levels of cell-cell junctions and ECM-cell adhesion in THBS1 KO cells, both in 3D and in vivo models. First, we generated tumor spheroids from WT and F5 cells (Fig. 4A). F5 cells formed spheroids with a reduced area compared to WT cells. Notably, similar results were obtained when WT-derived spheroids were generated in the presence of an integrin β1-blocking antibody. No spheroids were observed in the presence of a claudin-1-blocking antibody (Fig. 4A).

The impact of the THBS1 KO was further evaluated in xenograft models by assessing the tumorigenic potential of HuCCT-1 WT and F5 cells in BALB/c nude mice. To this end, single-cell suspensions of either WT or F5 cells were injected into the right and left flanks between the hind limbs and the dorsal region of 8-weeks-old BALB/c nude mice (Fig. 4B). Notably, no tumor masses were detected in mice injected with F5 cells 8 weeks after transplantation, whereas WT cells efficiently formed tumor (Fig. 4C-E).

### Exogenous administration of THBS1- and THBS2 increased integrin β1 activation in both epithelial and mesenchymal iCCA cell lines

**Fig. 5:**
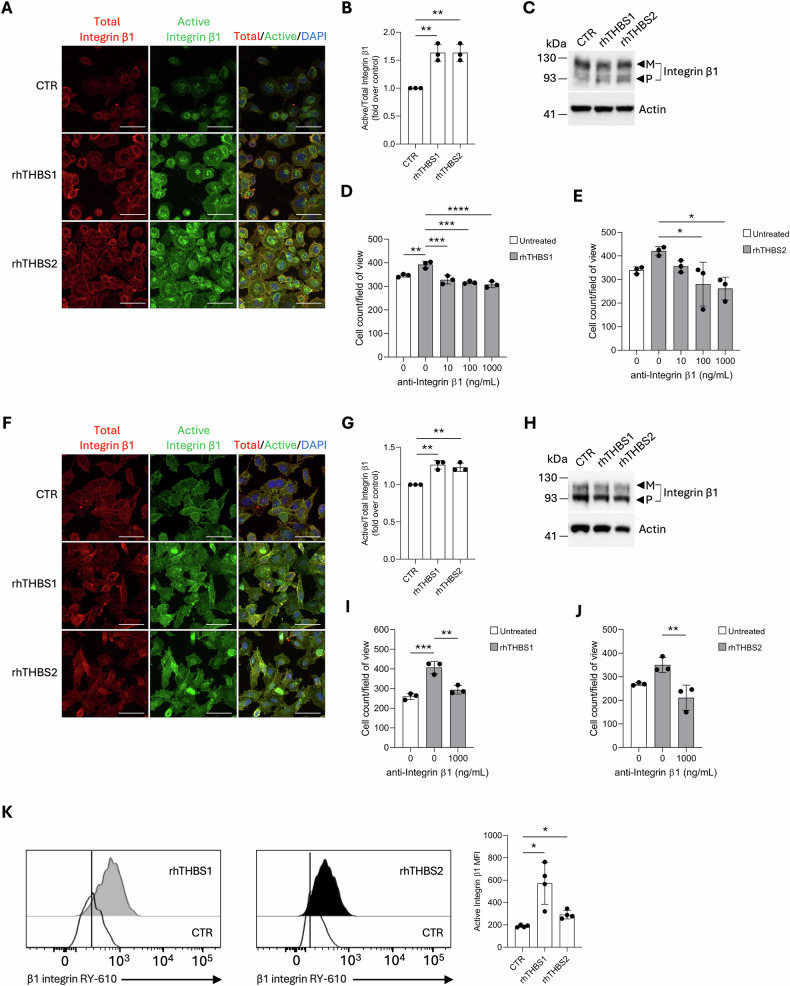
Exogenous THBS1 and THBS2 administration enhance integrin β1 activation in iCCA cells. IF analysis of total and active β1 integrin on HuCCT-1 (**A**) and CCLP1 (**F**) cells in response to rhTHBS1 and rhTHBS2 (1000 ng/mL). Quantitative analysis of the percentage of active with respect of total β1 integrin in HuCCT-1 (**B**) and CCLP1 (**G**) cells. Western blot analysis for β1 integrin on whole-cell extracts of HuCCT-1 (**C**) and CCLP1 (**H**) cells; actin was used as a loading control. Adhesion assay of HuCCT-1 cells in response to rhTHBS1 (**D**) and rhTHBS2 (**E**) (1000 ng/mL dose) and to increased concentrations of integrin β1 blocking antibody. Adhesion assay of CCLP1 cells in response to rhTHBS1 (**I**) and rhTHBS2 (**J**) (1000 ng/mL) in the presence of integrin β1 blocking antibody (1000 ng/mL). **K** Integrin β1 activation in HuCCT-1 cells treated either with rhTHBS1 or rhTHBS2 (1000 ng/mL) was analyzed by flow cytometry. Representative curves show mean fluorescence intensity (MFI) distributions for untreated cells (white), rhTHBS1-treated cells (gray), and rhTHBS2-treated cells (black). The bar graph shows the mean MFI from four independent biological replicates. Data are shown as mean ± SD from three biologically independent experiments unless otherwise indicated. Statistical significance was determined by one-way ANOVA with Dunnett’s (**B**, **G**) for comparisons to CTR and Tukey’s test for comparisons among all groups (**D**, **E**, **I**, **J**). When the assumption of equal variances was not met, Welch’s ANOVA was applied (**K**).*p < 0.05; **p < 0.01; ***p < 0.001; **** p < 0.0001.

In the TME of iCCA, our previous studies have demonstrated that THBS1 and THBS2, besides the expression in cancer cells, are predominantly expressed and released by CAFs [8]. Thus, it is conceivable that the effects of the THBSs autocrine positive feedback on integrin β1 activation in tumor cells are further enhanced by CAFs-mediated paracrine signaling on tumor cells. Consequently, both the HuCCT-1 and CCLP1 cells were treated with exogenous rhTHBS1 and recombinant human THBS2 (rhTHBS2) to assess the effects on the integrin β1 activation. Treatment with rhTHBS1 and rhTHBS2 induced integrin β1 activation, as demonstrated by the increased immunostaining for its activated form of integrin β1 compared to the untreated HuCCT-1 cells (Fig. 5A, B). Notably, rhTHBS1 and rhTHBS2 treatments did not affect integrin β1 expression (Fig. 5C). However, as expected, they enhanced the adhesive properties of the HuCCT-1 cells (Fig. 5D, E). Importantly, the anti-integrin β1 blocking antibody effectively counteracted the rhTHBS1- and rhTHBS2-induced increase in cell adhesion (Fig. 5D, E). All these findings were also confirmed in CCLP1 cells, indicating that the extracellular THBS1 and THBS2 contribute to shift from inactive to active state (Fig. 5F–J). We also investigated whether THBS1 and THBS2 were able to promote integrin β1 activation without interacting with the ECM. As shown in Fig. 5K, flow cytometry analysis showed an increase of integrin β1 activation after treatment with the THBSs, indicating that conformational activation is directly mediated by these MCPs.

### Integrin α3β1 and α6β1 mediate the THBS1- and THBS2-induced iCCA cell adhesion

**Fig. 6:**
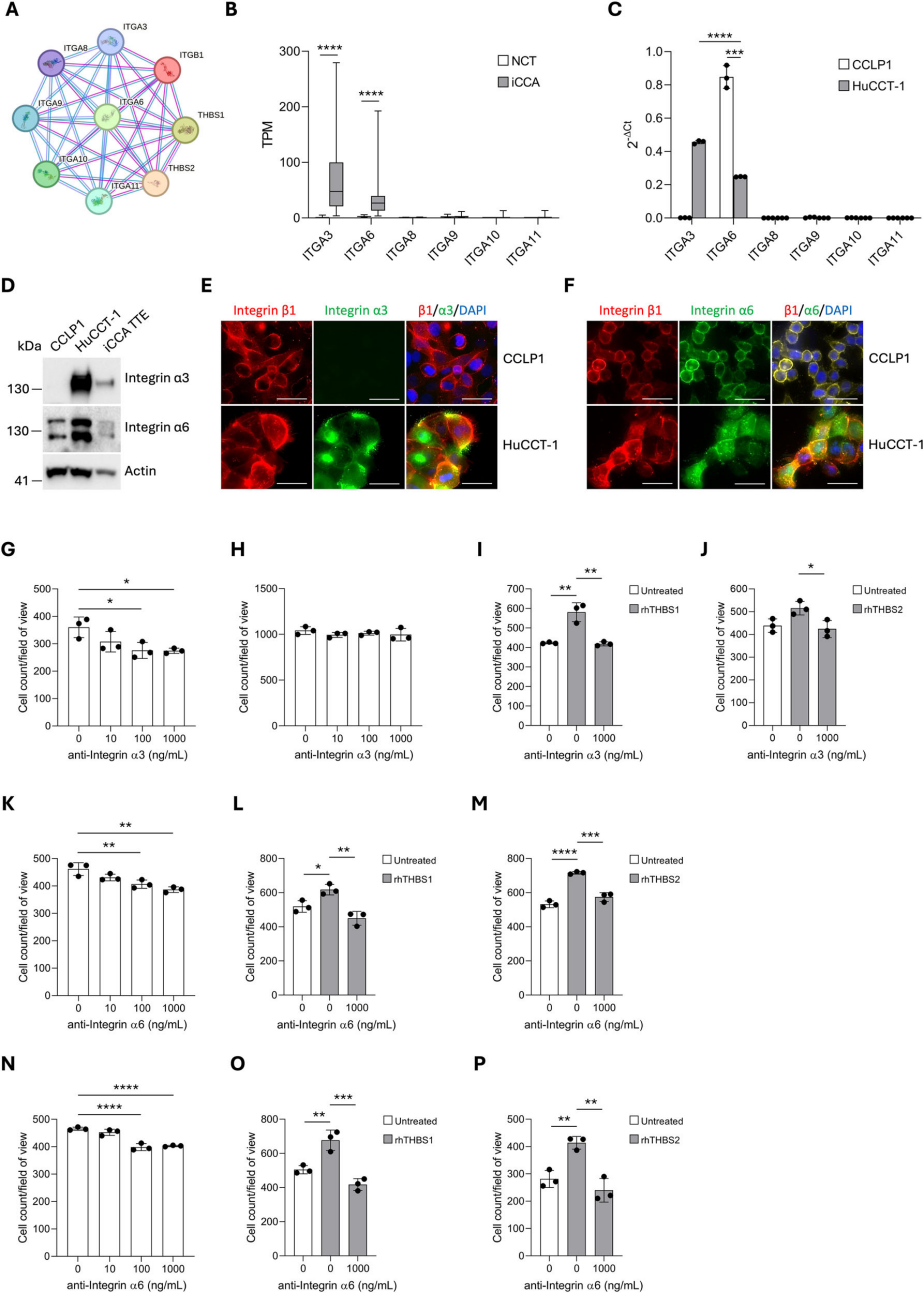
Integrin α3 and α6 are the α subunits involved in the THBS1- and THBS2-induced integrin β1 activation. **A** Protein-protein interaction network of THBS1, THBS2 and integrin β1 (query proteins) and experimentally determined interactors. **B** mRNA expression levels of the indicated genes in NCT and iCCA tissue specimens from iCCA patients were analyzed using GEO dataset GSE107943. Box plots show transcript abundance in TPM. **C** qPCR analysis of the indicated genes in CCLP1 and HuCCT-1 cells. Bar plots show 2^−ΔCt^ values normalized to GAPDH. **D** Protein expression levels of integrin α3 and integrin α6 in CCLP1 and HuCCT-1 cells assessed by immunoblotting. iCCA TTE and actin were used as positive and loading controls, respectively. Illustrative immunofluorescence images of HuCCT-1 and CCLP1 cells co-stained for integrin β1, integrin α3 (**E**) or integrin α6 (**F**). Scale bars, 50 μm. Adhesion assay of HuCCT-1 (**G**) and CCLP1 (**H**) cells upon treatment with integrin α3 blocking antibody at the indicated concentrations. Adhesion assay of HuCCT-1 cells in response to rhTHBS1 (**I**) and rhTHBS2 (**J**) (1000 ng/mL) in the presence of integrin α3 antibody. **K** Adhesion assay of HuCCT-1 cells upon treatment with integrin α6 blocking antibody at the indicated concentrations. Adhesion assay of HuCCT-1 cells in response to rhTHBS1 (**L**) and rhTHBS2 (**M**) (1000 ng/mL) in the presence of integrin α6 antibody. **N** Adhesion assay of CCLP1 cells upon treatment with integrin α6 blocking antibody at the indicated concentrations. Adhesion assay of CCLP1 cells in response to rhTHBS1 (**O**) and rhTHBS2 (**P**) (1000 ng/mL) in the presence of integrin α6 blocking antibody (1000 ng/mL). Bar plots represent mean ± SD of adhered cells from at least three independent experiments. P values were assessed using two-tailed Student’s t test for pairwise comparisons (**B**, **C**) and one-way ANOVA with Dunnett’s test for comparisons to untreated cells (**G**, **H**, **K**, **N**) and Tukey’s post hoc test for multiple group comparisons (**I**, **J**, **L**, **M**, **O**, **P**). *p < 0.05; **p < 0.01; ***p < 0.001; ****p < 0.0001.

Integrins are a family of 24 heterodimers formed by the combination of 18 α and 8 β subunits [39]. Understanding which specific quaternary structure of integrins is involved in the studied biological effect is essential for developing targeted therapeutic strategies. To identify integrin α subunits associated with the integrin β1 on the cell surface of the iCCA cells, we screened the potentially interacting proteins with integrin β1, THBS1 and THBS2 by using the STRING database (v11.5; https://string-db.org/cgi/input.pl) (Fig. 6A). Considering only experimental-based physical interactions, six potential α subunits were identified; their transcriptional expression levels were subsequently analyzed in silico on iCCA and NCT tissues (Fig. 6B) [24]. We found that integrin subunits α3 and α6 were exclusively expressed in cancer specimens. Consequently, we analyzed the transcriptional expression levels of integrins α3 and α6 in HuCCT-1 and CCLP1 cells (Fig. 6C). HuCCT-1 cells expressed both α3 and α6 subunits, while CCLP1 exclusively expressed the α6 subunit. These findings were confirmed by WB analysis (Fig. 6D) and IF (Fig. 6E, F). Therefore, we conducted an adhesion assay on HuCCT-1 cells in the presence of increasing concentrations of an anti-integrin α3-blocking antibody. A statistically significant decrease in cell attachment was observed, confirming that the integrin α3 is involved in promoting the cellular adhesive properties of the HuCCT-1 cells (Fig. 6G). As expected, no effect was observed in CCLP1 cells treated with anti-integrin α3-blocking antibody (Fig. 6H). The involvement of the integrin α3 in the THBS1- and THBS2-induced iCCA cell adhesion was also confirmed by treating HuCCT-1 with either rhTHBS1 or rhTHBS2. As expected, the anti-integrin α3-blocking antibody effectively counteracted the THBS1- and THBS2-induced increased cell adhesion in HuCCT-1 cells (Fig. 6I-J). Similar results were obtained for the integrin α6, both in HuCCT-1 (Fig. 6K–M) and CCLP1 (Fig. 6N–P) cell lines. These results show that integrin α3β1 and integrin α6β1 are engaged by the THBS1 and THBS2 on the cell surface of the iCCA cells.

### Antagonist THBS1-derived peptides interfere with the adhesive functions and spheroid formation of HuCCT-1 cells

**Fig. 7:**
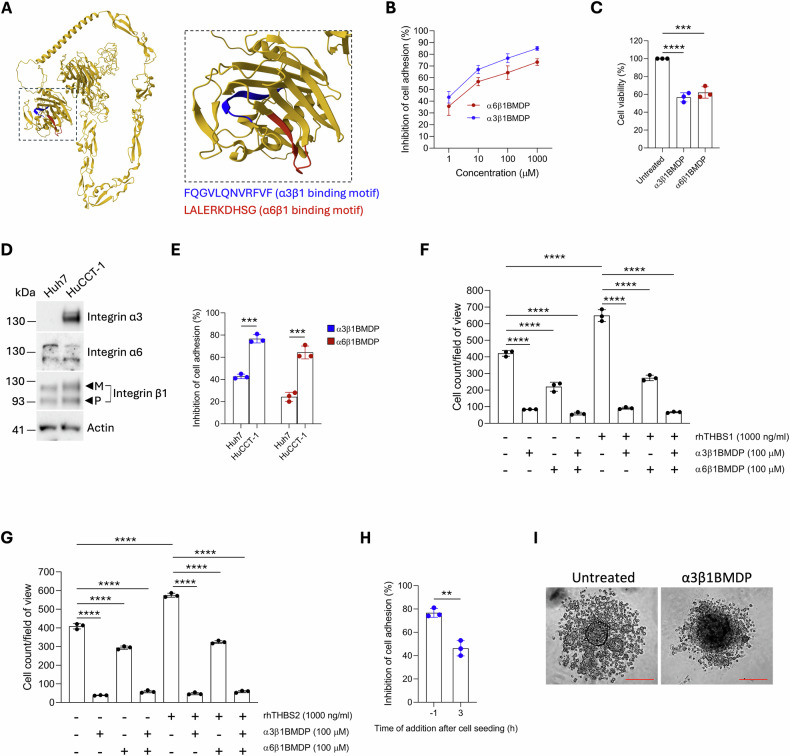
α3β1 and α6β1 BMDPs inhibit HuCCT-1 cell adhesion and hamper spheroids formation. **A** Monomeric structure of THBS1 highlighting the N-terminal globular domain; the α3β1 (FQGVLQNVRFVF) and α6β1 (LALERKDHSG) binding motifs sequences are indicated. Created with Alphafold.ebi.ac.uk with granted permission and license. The inset shows a close-up of the region where the two binding motifs are in close proximity. **B** Percentage of cell adhesion inhibition in HuCCT-1 cells following treatments with α3β1 or α6β1 BMDP at the indicated concentrations; each point represents a biological triplicate. **C** MTT cell viability assay of HuCCT-1 cells at 100 µM dose of either α3β1 or α6β1 BMDP; values are shown relative to untreated control. **D** Immunoblot analysis of integrin α3, integrin α6, and integrin β1 in Huh7 and HuCCT-1 cells. Actin was used as a loading control. **E** Percentage of cell adhesion inhibition in Huh7 and HuCCT-1 cells following treatments with α3β1 or α6β1 BMDP at 100 µM dose; each point represents a biological triplicate. Cell adhesion assay of HuCCT-1 cells exposed to 1000 ng/mL rhTHBS1 (**F**) or rhTHBS2 (**G**), alone or with 100 µM of α3β1 or α6β1 BMDPs. **H** Percentage of HuCCT-1 cell adhesion inhibition following treatments with α3β1 BMDP (100 µM dose) added 1 h before seeding (–1) and 3 h post-seeding (3). **I** Representative brightfield images of spheroids treated or untreated with 100 µM of α3β1 BMDP. Scale bars, 200 µm. Data are mean ± SD from three independent experiments. Statistical significance was determined using two-tailed Student’s t test (**E**, **H**) or one-way ANOVA with Dunnett’s test (**C**) and Tukey’s post hoc test for multiple comparisons (**F**, **G**). **p < 0.01; ***p < 0.001; ****p < 0.0001.

Considering all the collected data, we believe that one of the most valuable approaches to counteract the oncogenic function of THBS1 and THBS2, regardless of their cell origin, is to directly interfere with their binding to integrins α3β1 and α6β1. Since specific inhibitors or antagonists targeting the α3β1 and α6β1 integrins are still unavailable, we hypothesize that peptides derived from the THBS1- and THBS2-specific linear amino acid motifs, known to bind these integrins, could serve as potent and specific antagonists. The binding site of THBS1 with α3β1 integrin has been mapped to residues 175-242. The THBS1 peptide FQGVLQNVRFVF, corresponding to residues 190-201, is a potent inhibitor of THBS1-induced cell adhesion [40]. Additionally, the peptide LALERKDHSG has been identified as an α6β1-binding sequence in THBS1 [41]. This peptide, located at residues 87-96 in the N-terminal globular domain, inhibits α6β1-dependent cell adhesion to both THBS1 and, most importantly, to THBS2 at a concentration of 200 µM [41]. To note, by analyzing the THBS1 protein structure [42], both the α3β1 and α6β1 binding sites were found to be proximally located within the N-terminal globular domain (Fig. 7A). Thus, both these integrin β1 binding motif-derived peptides (BMDPs) were selected as potential competitive inhibitors of integrin α3β1 and α6β1. Both α3β1 and α6β1 BMDPs were individually preincubated for 1 h at increasing concentrations with HuCCT-1 cells before seeding for cell adhesion assays. As shown in Fig. 7B, we observed a dose-dependent effect in inhibiting cell adhesion, noting that the α3β1 BMDP performed higher inhibitor efficiency compared to α6β1 BMDP by inhibiting the adhesiveness in three-quarters of the cell population already at 100 μM dose. We also assessed the cell viability of HuCCT-1 in response to peptide administration at 100 μM dose. As expected, the inhibition of cell adhesion in treated cells led to reduced levels of cell viability (Fig. 7C). We next examined whether α3β1 and α6β1 BMDPs were able to specifically target iCCA cells within the heterogeneous liver cell population. As shown in Fig. 1B, the α3 subunit was found to be exclusively expressed in the malignant cholangiocytes. Thus, the cell adhesion assays in response to either α3β1- or α6β1 BMDP administration at 100 μM dose were performed on Huh7 cells, a well-established HCC cell line which expresses only the α6 subunit (Fig. 7D). As expected, both α3β1 and α6β1 BMDPs exhibited lower inhibitor efficiency compared to that observed on the HuCCT-1 cells (Fig. 7E). We then tested α3β1 or α6β1 BMDP on the ability to interfere with the exogenous THBS1- and THBS2-induced iCCA cell adhesion. We found that 100 μM dose of both the α3β1- or α6β1 BMDP were able to counteract the rhTHBS1- and rhTHBS2-induced cell adhesiveness of HuCTT-1 cells (Fig. 7F-G). Once more, α3β1 BMDP showed higher efficiency in inhibiting cell adhesion. Similar results were obtained also on CCLP1 cells, thus demonstrating the efficacy of the integrin β1 BMDPs in interfering with the α6β1 integrin function on mesenchymal cells (Supplementary Fig. 4A-B). Subsequently, we assessed the efficacy of α3β1 BMDP to physically displace cancer cells from the ECM. α3β1 BMDP added 3 h after HuCCT-1 cell seeding was able to detach 50% of adherent cells (Fig. 7H). Finally, we tested the effects of α3β1 BMDP to interfere with the HuCCT-1-derived spheroid formation. As shown in Fig. 7I, α3β1 BMDP significantly hampered the spheroid formation, thus highlighting its potential in counteracting the iCCA cell malignant behavior.

## Discussion

In this study, we investigated the role of THBS1 in the regulation of cell-to-cell adhesion and cell-to-ECM interaction in iCCA. Our results demonstrate that THBS1 expression and release are pivotal for coordinating these interactions. In this context, we identified two major molecular events underlying THBS1-driven iCCA pathobiology. First, THBS1, together with THBS2, mediates the iCCA cell-ECM adhesion by engaging α3β1 and α6β1 integrins and promoting their transition from inactive to active conformation. Second, THBS1 expression is required for the establishment of cell-cell junctions and the maintenance of tightly apposed cellular membranes. Collectively, these findings provide new mechanistic insights into THBS1 function in iCCA and may pave the way for developing novel targeted therapeutic strategies in the management of iCCA.

To date, surgical resection or liver transplantation for cholangiocarcinoma patients diagnosed at an early stage remain the only potentially curative options [43]. In advanced disease, the current first-line treatment, a combination of chemotherapy (gemcitabine and cisplatin) and immunotherapy (i.e., durvalumab or pembrolizumab), shows suboptimal outcomes with 2-year survival rates rarely exceeding 20% [43]. Thus, more effective and targeted therapies are urgently required. In recent years, integrins α3, α6, and β1 emerged as important players in the malignant transformation of the iCCA cells, thus highlighting them as attractive therapeutic targets [44, 45]. Here, we demonstrated the presence of α3β1 and α6β1 heterodimers on the plasma membrane and directly connected their oncogenic activity to that of THBS1 and THBS2. Moreover, by using blocking antibodies and inhibiting peptides targeting the interaction sites between THBS1 and α3β1 and α6β1 heterodimers, we disrupted integrin function by preventing THBSs-induced activation both in epithelial-like (HuCCT-1) and mesenchymal-like (CCLP1) iCCA cell lines. To date, none of the integrin antagonists proposed for preclinical cancer studies have specifically targeted α3β1 and α6β1 heterodimer functions [46]. Since α3β1 and α6β1 integrins are mostly expressed by malignant cholangiocytes, antibodies and blocking peptides to specifically target these integrins may represent specific and effective therapeutic tools for iCCA.

It is well known that the success of integrin antagonists in preclinical cancer models has not been completely reflected in clinical settings [46, 47]. This largely depends on variable integrin expression and redundancy in their function, as well as on the distinct roles that integrins exert at various disease stages [46]. Here, besides the pivotal role in the autocrine and paracrine activation of α3β1 and α6β1 integrins, we reveal a novel function of THBS1 in iCCA cells that could be exploited therapeutically. Indeed, the loss of THBS1 expression in HuCCT-1 cells markedly impaired the establishment of tightly apposed intercellular contacts, leading to structurally lax and mechanically unstable junctions. Concomitantly, the reduced adhesion to ECM weakens epithelial cancer cell monolayers and spheroids and this likely explains why no tumor masses developed in THBS1 KO F5 cell xenografts. Moreover, the reduced cell-to-cell and cell-to-ECM interaction observed in THBS1 KO cells occurred without (i) triggering the EMT program, (ii) increasing cell motility, and (iii) upregulating metalloproteinase expression. Thus, loss of THBS1 expression contrasts with the development of a more malignant phenotype. In this context, we propose that upstream epigenetic elements, such as miRNAs, controlling post-transcriptional expression of THBS1 and THBS2 could be therapeutically exploited. Specifically, nanoparticle loaded with specific tumor-suppressor miRNAs and delivered to the tumor site via α3β1 and α6β1 integrin binding ligands may modulate THBS1 expression and inhibit tumor progression.

In conclusion, our findings unveiled novel insights on the role of THBS1 in ensuring the correct formation of iCCA cell junctions and promoting cell adhesiveness to substrate via α3β1 and α6β1 integrins. Furthermore, we provide compelling evidence that antibodies and peptides interfering with α3β1 and α6β1 integrin activation may represent a promising therapeutic strategy for cancer-targeted therapy in iCCA and potentially other solid tumors, such as breast cancer and small cell lung carcinoma, where THBS1 and THBS2 exert oncogenic functions by engaging these integrins.

## Methods

### Cell lines and cell culture

Human iCCA cell lines CCLP1 and HuCCT-1 were kindly provided by Prof. D. Alvaro (Sapienza University of Rome) and Prof. F. Marra (University of Florence), respectively. CCLP1 cells display a mucin-negative mesenchymal phenotype, whereas HuCCT-1 cells are mucin-positive and epithelial in nature [48]. Both cell lines were cultured in RPMI-1640 medium (Gibco, Thermo Fisher Scientific) supplemented with 10% fetal bovine serum (FBS; Euroclone), 2 mM L-glutamine (Lonza) and 1% penicillin–streptomycin (Gibco). Cells were maintained at 37 °C in a humidified atmosphere containing 5% CO₂. Both cell lines were regularly tested for mycoplasma contamination.

### Patients and tumor samples

The study was approved by the Institutional Ethic Committee of Sapienza University of Rome (prot. 756/18, Rif. 5172) and conforms to the ethical guidelines of the 1975 Declaration of Helsinki. Written informed consent was obtained from all patients prior to study participation. All samples were fully anonymized, and only clinical information relevant to the study was retained. Inclusion criteria for cancer patients were a histopathological confirmed diagnosis and availability of clinical data. Exclusion criteria included mixed tumors, presence of other malignancies, and uncertain diagnosis of iCCA, pCCA, or HCC.

### Recombinant proteins and synthetic peptides

CCLP1 and HuCCT-1 cells were treated with rhTHBS1 (Sigma Aldrich) and rhTHBS2 (R&D Systems Europe Ltd., Abingdon, Oxfordshire, UK) proteins or with synthetic α6β1BMDP and α3β1BMDP (Thermo Scientific Custom Peptide Synthesis Service). Cellular functional assays and molecular analyses were performed using rhTHBS1 and rhTHBS2 at a final concentration of 1000 ng/mL, as previously described [9]. Synthetic peptides ( > 98% purity; Fmoc chemistry) were tested individually or in combination at concentrations of 1, 10, 100 and 1000 µM. These concentrations were selected based on previous studies [41, 49] with modified dose ranges.

### Analysis of public bulk and single-cell RNA-seq datasets

Bulk RNA-sequencing data were retrieved from the Gene Expression Omnibus (GEO) under accession GSE107943 [24], including 30 iCCA tumor samples and matched non-tumoral tissues. Expression levels of ITGB1, ITGB3, CD36, CD47, and integrin alpha subunits (ITGA3, ITGA6, ITGA8, ITGA9, ITGA10, and ITGA11) were extracted as transcripts per million (TPM), and mean expression values were used for graphical representation. TPM values were obtained directly from the GEO analysis interface without additional processing or transformation. Publicly available single-cell RNA-sequencing (scRNA-seq) datasets were obtained from GEO under accession numbers GSE151530 [25], and GSE138709 [26]. Raw count matrices were imported into R and analyzed using the Seurat package v5.2.1 [50]. Datasets were integrated using RPCA-based integration method. For downstream analyses, principal components 1–10 from the integrated RPCA reduction were used. Clustering was performed using a resolution parameter of 0.3.

### Protein–protein interaction network analysis

Protein–protein interaction networks were generated using the STRING database by querying ITGB1, THBS1 and THBS2 as seed proteins. Only interactions supported by experimental evidence or curated databases were considered. The network was restricted to a maximum of five first-shell interactors to identify candidate integrin alpha subunits.

### Immunoblotting

For immunoblot analysis, tissues or cells were lysed for 30 min on ice using Cell Extraction Buffer (CEB; Thermo Fisher Scientific, FNN0011) supplemented with a protease inhibitor cocktail (Sigma, P8340), according to the manufacturer’s instructions. Protein concentration was determined using a bicinchoninic acid (BCA) assay (Thermo Fisher Scientific, 23250). Equal amounts of protein (10 µg per sample) were denatured and separated by sodium dodecyl sulfate–polyacrylamide gel electrophoresis (SDS–PAGE) using 4–12% Bolt™ Bis-Tris Plus Mini Protein Gels (Invitrogen, NW041) and MOPS SDS running buffer (Invitrogen, B0001). Proteins were transferred onto nitrocellulose membranes, and transfer efficiency and loading uniformity were verified by reversible Ponceau S staining. Membranes were blocked with 5% skim milk in phosphate-buffered saline (PBS) containing 0.1% Tween 20 and incubated with primary antibodies, followed by horseradish peroxidase (HRP)-conjugated secondary antibodies. Immunoreactive signals were detected using Luminata™ Western HRP chemiluminescent substrates (Millipore). HRP-conjugated secondary antibodies (Jackson ImmunoResearch) were used at a 1:5000 dilution. Chemiluminescent signals were acquired using a ChemiDoc™ Touch Imaging System (Bio-Rad), and band intensities were quantified using Image Lab software v5.1.2 (Bio-Rad). A detailed list of primary antibodies is provided in Supplementary Table 4.

### RNA isolation and real-time qPCR

Total RNA was extracted from CCLP1 and HuCCT-1 cells using TRIzol reagent (Thermo Fisher Scientific) according to the manufacturer’s instructions. RNA quantity and purity were assessed using NanoDrop One and Qubit instruments (Thermo Fisher Scientific). Primers were designed using Primer-BLAST (http://www.ncbi.nlm.nih.gov/tools/primer-blast, accessed June 18 and October 6, 2025). Real-time quantitative PCR (qPCR) was performed using the Power SYBR Green RNA-to-CT™ 1-Step Kit (Thermo Fisher Scientific) with 20 ng of total RNA per reaction. Reactions were run on a 7900HT Fast Real-Time PCR System (Thermo Fisher Scientific). THBS1 and THBS2 expression levels in iCCA cell lines were compared with commercially available human ovary RNA (Ambion®), used as a positive control for high transcript expression [51]. qPCR data were analysed using SDS RQ Manager software v1.2 (Thermo Fisher Scientific). Relative gene expression levels were calculated using the 2^⁻ΔCt^ method, with GAPDH used as the reference gene for normalization. Primer sequences are provided in Supplementary Table 5.

### THBS1 KO by CRISPR/Cas9 and RNA-sequencing

A Cas9-expressing plasmid was obtained from Addgene (plasmid #62988). Single-guide RNA (sgRNA) targeting the human THBS1 gene (sequence: CCTGTCTAACAGAGTCTGG) was designed using the Synthego web tool and cloned as annealed duplex oligonucleotides into the Cas9 vector, as previously described [52]. Correct insertion of the sgRNA sequence was confirmed by Sanger sequencing. HuCCT-1 cells were transfected with the CRISPR/Cas9 construct using Lipofectamine™ 3000 (Thermo Fisher Scientific) according to the manufacturer’s instructions. Thirty-six hours after transfection, Cas9-expressing cells were selected using puromycin (1,5 µg/mL). After 48 h of selection, surviving cells were serially diluted and seeded into 96-well plates to obtain monoclonal populations. Individual clones were screened for THBS1 KO by immunoblotting, and editing efficiency was evaluated at the protein level. HuCCT-1 cells served as the parental wild-type (WT) control for CRISPR/Cas9-derived knockout clones (W8 and F5) and are hereafter referred to as WT when analyzed in parallel with knockout cells. To assess transcriptional changes associated with THBS1 KO, total RNA from WT and F5 was extracted and subjected to RNA-sequencing. RNA-seq was performed on three independent biological replicates per condition. Raw FASTQ files were quality-checked with FastQC v0.12.1. Reads were aligned to the human genome (hg38) using HISAT2 [53], and duplicate reads were removed with Samblaster v0.1.26 [54]. Mapped reads were summarized into gene-level counts using htseq-count v2.0.3 [55] with gencode V49 [56] primary assembly annotation (mode = intersection-nonempty, stranded = reverse, id = gene_name). Raw counts were imported into R and analyzed using the DESeq2 package v1.44.0 [57] to identify differential gene expression.

### MTT cell viability assay

Cells were manually counted using a hemocytometer and seeded in 96-well plates at a density of 5 x 10³ cells per well. Cell viability of WT, W8, and F5 cell lines was assessed using a colorimetric MTT assay (CyQUANT™ MTT Cell Viability Assay, V13154; Invitrogen), as previously described [58]. After 24, 48, and 72 h, MTT reagent was added and plates were incubated to allow for formazan crystal formation. Crystals were subsequently solubilized, and absorbance was measured at 570 nm. To evaluate the effects of α6β1BMDP and α3β1BMDP on HuCCT-1 cell viability, cells were treated with peptides (100 µM) before seeding and plated in 96-well plates. Cell viability was assessed 24 h after seeding by MTT assay as described above.

### Spheroid culture and formation assay

Spheroid culture conditions for WT and F5 cells were established as previously described [59]. Briefly, cells were suspended in DMEM/F12 medium (Invitrogen, USA) supplemented with 1x B-27 Serum-Free Supplement (Invitrogen, USA), 1% penicillin–streptomycin, 0.01 µg/mL epidermal growth factor (EGF), and 0.01 µg/mL basic fibroblast growth factor (bFGF) (both Invitrogen, USA). Cells were seeded at a density of 5 x 10³ cells per well in ultra-low attachment 96-well plates (Corning, USA). For antibody treatment experiments, WT cells were cultured in the presence of either an anti–integrin β1 antibody or an anti–claudin-1 antibody (1000 ng/mL). In a separate set of experiments, HuCCT-1 cells were treated with the α3β1BMDP peptide (100 µM) before spheroid formation and cultured under the same conditions. After 7 days of culture, spheroids were imaged using a Zeiss Primovert inverted microscope (Carl Zeiss, Oberkochen, Germany) equipped with a 10× objective. Images were acquired using ZEN 2.0 software. Spheroid area was quantified for WT and F5 cells using ImageJ/Fiji software.

### Cell adhesion assay

Cell adhesion assays were conducted to assess basal adhesive capacity, the effects of integrin-blocking antibodies or peptides, and the ability of these treatments to counteract thrombospondin-induced adhesion. A thin layer of Matrigel (2,5 µg per well; Corning® Matrigel® Growth Factor Reduced (GFR) Basement Membrane Matrix, Corning, NY, USA) was dispensed into 48-well plates under chilled conditions and incubated for 1 h at 37 °C to allow polymerization. iCCA, W8, and F5 cell lines were seeded at a density of 2.5 x 10⁴ cells per well in complete medium and allowed to adhere for 45 min at 37 °C. For functional inhibition assays, cells were pre-incubated in microtubes for 1 h at room temperature with the indicated concentrations (10, 100 or 1000 ng/mL) of anti–integrin β1, anti–integrin α3, or anti–integrin α6 antibodies before seeding. In parallel experiments, iCCA cell lines were treated with α6β1BMDP or α3β1BMDP at concentrations of 1, 10, 100, or 1000 µM. To evaluate whether antibody- or peptide-mediated treatments could counteract the pro-adhesive effects of thrombospondins, rhTHBS1 and rhTHBS2 were added at a final concentration of 1000 ng/mL. Cells were then incubated for an additional 45 min at 37 °C. Non-adherent cells were removed by shaking the plates at 500 rpm for 15 s, followed by gentle washing with 1x PBS. Adherent cells were fixed in 100% ice-cold methanol for 15 min at −20 °C and stained with 0.1% crystal violet (Santa Cruz Biotechnology, Dallas, TX, USA; sc-207460) for 15 min at room temperature in the dark. After two washes with deionized water, the plates were air-dried. Adherent cells were imaged by acquiring five random optical fields per well using a Zeiss Primovert inverted microscope (Carl Zeiss, Oberkochen, Germany) equipped with a 4× objective. Images were acquired using ZEN 2.0 software. The number of adherent cells was quantified by direct counting using ImageJ v1.54. Results are expressed as the mean number of adherent cells per five optical fields from three independent biological replicates.

### siRNA-mediated ITGB1 gene silencing

For small interfering RNA (siRNA) experiments, HuCCT-1 cells were seeded at a density of 2×10⁵ cells per well in 24-well plates 24 h prior to transfection. Transfection was performed using Lipofectamine RNAiMAX Reagent (Thermo Fisher Scientific) according to the manufacturer’s instructions. Briefly, scramble control siRNA or two independent siRNAs targeting ITGB1 (siITGB1#1 and siITGB1#2), obtained from Sigma-Aldrich (Merck), were diluted in Opti-MEM Reduced Serum Medium (Gibco, Life Technologies) to a final concentration of 50 nM. In parallel, Lipofectamine RNAiMAX was diluted in Opti-MEM and incubated for 5 min at room temperature. The diluted siRNA and Lipofectamine RNAiMAX were then combined and incubated for 15–20 min at room temperature to allow complex formation. The siRNA–lipid complexes were added dropwise to the cells, which were maintained under standard culture conditions. Cells were incubated for 48 h prior to protein extraction, and transfection efficiency was assessed by Western blot analysis.

### Wound healing assay

WT and F5 cells were seeded in 12-well plates and cultured to confluence in complete RPMI-1640 medium. Upon reaching confluence, cells were serum-starved overnight in RPMI-1640 medium supplemented with 0,2% FBS. A linear wound was generated using a sterile 200 µl pipette tip. Cells were gently rinsed to remove debris and maintained in low-serum medium throughout the assay.

Images of the wound area were acquired immediately after scratching (T0) and after 10 h using a Zeiss Primovert inverted microscope (Carl Zeiss, Oberkochen, Germany) equipped with a 4× objective. Image acquisition was performed using ZEN 2.0 software. Residual wound area was quantified using ImageJ v1.54 and expressed as the percentage of wound closure normalized to the T0 time point.

### Xenograft tumor model

The impact of THBS1 KO on tumorigenicity was evaluated using a xenograft mouse model by comparing WT and F5 cells. All animal procedures were approved by the Italian Ministry of Health and conducted in compliance with Legislative Decree no. 26/2014 on the protection of animals used for scientific purposes. Male BALB/c nude mice (8 weeks old; *n* = 4) were purchased from Charles River Laboratories (Wilmington, MA, USA) and housed under standard conditions in accordance with institutional animal care guidelines. The sample size for animal experiments was based on previous studies and experimental feasibility [8]. No randomization was performed, since single-cell suspensions containing 1 x 10⁶ WT or F5 cells were injected subcutaneously into opposite flanks of the same mouse (left and right flanks, respectively), between the hind limb and the dorsal region. Tumor growth was monitored weekly by caliper measurements. No blinding was performed during the experiment or outcome assessment. Tumor volume was calculated using the formula: *(length x width²) x 0,5*. After 8 weeks, mice were euthanized by CO_2_ inhalation, and tumors were excised and fixed in 10% neutral-buffered formalin for downstream analyses.

### Immunofluorescence microscopy

HuCCT-1 and CCLP1 iCCA cell lines, as well as THBS1 KO subclones (W8 and F5), were seeded onto glass chamber slides (Falcon Culture Slides) and cultured for 24 h. Cells were washed twice with PBS and fixed with 4% paraformaldehyde for 20 min at room temperature. For claudin-1 immunostaining, including claudin-1/THBS1 co-immunofluorescence experiments, cells were fixed with ice-cold 100% methanol as previously described [60]. Fixation-dependent differences in THBS1 immunoreactivity compared with paraformaldehyde fixation are shown in Supplementary Fig. 1b. Following fixation, cells were permeabilized with 0.1% Triton X-100 in PBS for 15 min at room temperature and blocked for 1 h with 1% bovine serum albumin (BSA) in PBS to prevent non-specific antibody binding. Primary antibodies (listed in Supplementary Table 4) were diluted 1:50 or 1:100 in blocking solution and incubated overnight at 4 °C. After three washes with PBS, cells were incubated with fluorophore-conjugated secondary antibodies at a dilution of 1:500 in blocking solution and incubated for 1 h at room temperature in the dark. Secondary antibodies used for immunostaining were Alexa Fluor™ 647 anti-mouse (Invitrogen), Alexa Fluor™ 594 anti-mouse (Invitrogen), Alexa Fluor™ 488 ant-rabbit (Invitrogen), Cyanine Cy™3 anti-rat (Jackson ImmunoResearch). Cell nuclei were counterstained with DAPI diluted 1:1000, by incubation for 10 min in the dark. After three additional washes in PBS, slides were mounted with coverslips before imaging. The filamentous actin cytoskeleton was stained using Alexa Fluor™ 647 Phalloidin (1:400; Invitrogen). Morphometric analyses of stress fiber density, fiber length, and cell area were performed using Fiji software. Stress fiber density and average fiber length were quantified from the same images, with measurements calibrated to the scale bar. Images were converted to 8-bit grayscale, the background was subtracted, and the contrast was enhanced. A global automatic threshold (Otsu, dark background) was applied to generate a binary mask of actin filaments. Stress fiber density was calculated as the percentage of phalloidin-positive pixels relative to the total image area. Cell area was calculated for each field of view using the formula: Cell Area=Area of the Field of View/Number of Cells in the Field. All images were analyzed using identical parameters to ensure comparability between WT and F5 cells. Co-immunofluorescence experiments to assess integrin β1 activation were performed using a total integrin β1 antibody (clone 12G10) in combination with an antibody specific for the active conformation of integrin β1 (clone 9EG7) [61]. These experiments were conducted both under basal conditions (24 h culture) and after 45 min of matrix adhesion. For the latter, glass slides were coated with Matrigel® (Growth Factor Reduced (GFR) Basement Membrane Matrix; Corning) and seeded with the cells. Where indicated, cells were seeded in the presence or absence of rhTHBS1 or rhTHBS2 (1000 ng/mL) and incubated for 45 min at 37 °C in a humidified atmosphere containing 5% CO₂. At the end of the treatment, cells were then fixed with 4% paraformaldehyde and processed for immunofluorescence. Images were acquired using a Celldiscoverer 7 confocal microscope (Carl Zeiss AG) with a 50× objective in Z-stack mode, 20x objective in brightfield (transmitted light) images, or a Zeiss Axio Zoom inverted microscope with a 40× objective, using ZEN Blue or ZEN 2.0 software (Carl Zeiss AG). Total fluorescence intensity (TFI; arbitrary units) was quantified exclusively for total integrin β1 in HuCCT-1 and CCLP1 cells as the integrated sum of pixel intensities within regions of interest (ROIs) using Fiji software. ROIs were defined by thresholding to include all positive pixels within each field. The resulting values represent the integrated sum of pixel intensities within the ROIs, providing a measure of total fluorescence signal. The relative fraction of active integrin β1 over total integrin β1 was quantified across the indicated experimental conditions using ZEN Blue software (Carl Zeiss AG) by threshold-based analysis. For each condition, 12 microscopic fields were analyzed per experiment from three independent biological replicates. All images were acquired using identical laser power and exposure settings.

### Flow cytometric analysis of integrin β1 activation following rhTHBS1 and rhTHBS2 stimulation

HuCCT-1 cells were detached using Versene and washed with PBS containing 5 mM EDTA. Cells were then incubated with or without rhTHBS1 or rhTHBS2 (1000 ng/mL) for 1 h at 37°C, in the presence of the primary antibody HUTS21 (a kind gift from Dr Carlos Cabañas), which recognizes the active conformation of integrin β1 [62]. Stimulation was stopped by addition of ice-cold PBS, and cells were subsequently labeled with Alexa Fluor™ 594-conjugated anti-mouse secondary antibody (Invitrogen) for 20 min on ice in the dark. After washing, samples were acquired on a BD FACSSymphony A5 flow cytometer (BD Biosciences, San Jose, CA, USA) and analyzed using FlowJo v9.2.3 (Tree Star, Ashland, OR, USA). Integrin β1 activation was quantified as the mean fluorescence intensity (MFI) of HUTS21 staining.

### Statistical analysis

Statistical analyses were performed using GraphPad Prism v8 (GraphPad Software, Boston, MA, USA). Data distribution was assessed for normality using the Shapiro–Wilk test. Non formal power calculation was performed; sample size was based on previous experience and literature in the field. Non samples or animal were excluded from the analysis. For comparisons between two groups, a two-tailed unpaired Student’s *t*-test was used. For multiple-group comparisons, one-way analysis of variance (ANOVA) or Welch’s ANOVA was performed, followed by Dunnett’s post hoc test for comparisons with control groups or Tukey’s post hoc test for all pairwise comparisons, as appropriate. Data are presented as mean ± standard deviation (SD) from at least three independent biological replicates. Statistical significance was defined as *p* < 0.05.

## Supplementary information

Table S1

Supplementary Figures and Tables S2-S5

Uncropped blot and gel images

## Data Availability

The datasets generated during and/or analyzed during the current study are available in the NCBI repository, [https://dataview.ncbi.nlm.nih.gov/object/PRJNA1381298?reviewer=8m7p0b4g68id1sj6gt6fu9upld.].
