# Supplementary material for: Disruption of thrombospondin 1/2-integrin β1 axis impairs cell adhesion and tumor growth in intrahepatic cholangiocarcinoma
Source: Cell Death Discov. 2026 Jun 1;12:323. doi: 10.1038/s41420-026-03164-1 (PMC13438543; doi:10.1038/s41420-026-03164-1)
Supplement: Supplementary file 1 [file 41420_2026_3164_MOESM2_ESM.pdf]

Disruption of thrombospondin 1/2-integrin β1 axis impairs cell adhesion and tumor growth in intrahepatic cholangiocarcinoma

Running title: THBS1/2-ITGB1 axis primes iCCA growth

Veronica Porreca[1], Ludovica Giancola[1], Luca Sallustio[1], Alessia Conigliaro[1], Pietro Angelone[1], Roberta Marrone[1], Francesco Greco[1], Dionino Marco Giangrande[1], Giulio Bontempi[1,2], Michele Stella[3], Biagio Palmisano[1], Giuseppina Mignogna[4], Martina Meucci[5], Fabio Melandro[6], Gianluca Mennini[6], Massimo Rossi[6], Mara Riminucci[1], Alessandro Corsi[1], Helena Stabile[1], Marco Ragusa[3], Antonio Filippini[5], Valerio Fulci[1], Raffaele Strippoli[1,2], Bruno Maras[4], Carmine Mancone[1*]

Supplementary Information – Text Summaries

Figure S1: immunoblot of iCCA patient samples and THBS1 immunofluorescence in HuCCT-1 cells.

Figure S2: integrin β1 immunofluorescence in W8 cells after 1000 ng/mL rhTHBS1

Figure S3: RNA-seq, functional assays, integrin β1/Rab5 co-immunostaining in WT and F5 cells, immunoblot analysis of total and phosphorylated FAK in HuCCT-1 cells treated with 1000 ng/mL anti-integrin β1 antibody, siRNA ITGB1 in HuCCT-1 cells.

Figure S4: Dose-dependent inhibition of CCLP1 cell adhesion by α3β1 or α6β1 BMDPs, effects of rhTHBS1 or rhTHBS2, alone or in combination with α3β1 or α6β1 BMDPs, on CCLP1 cell adhesion.

Tables S1–S5 and full-length uncropped Western blots are also provided.

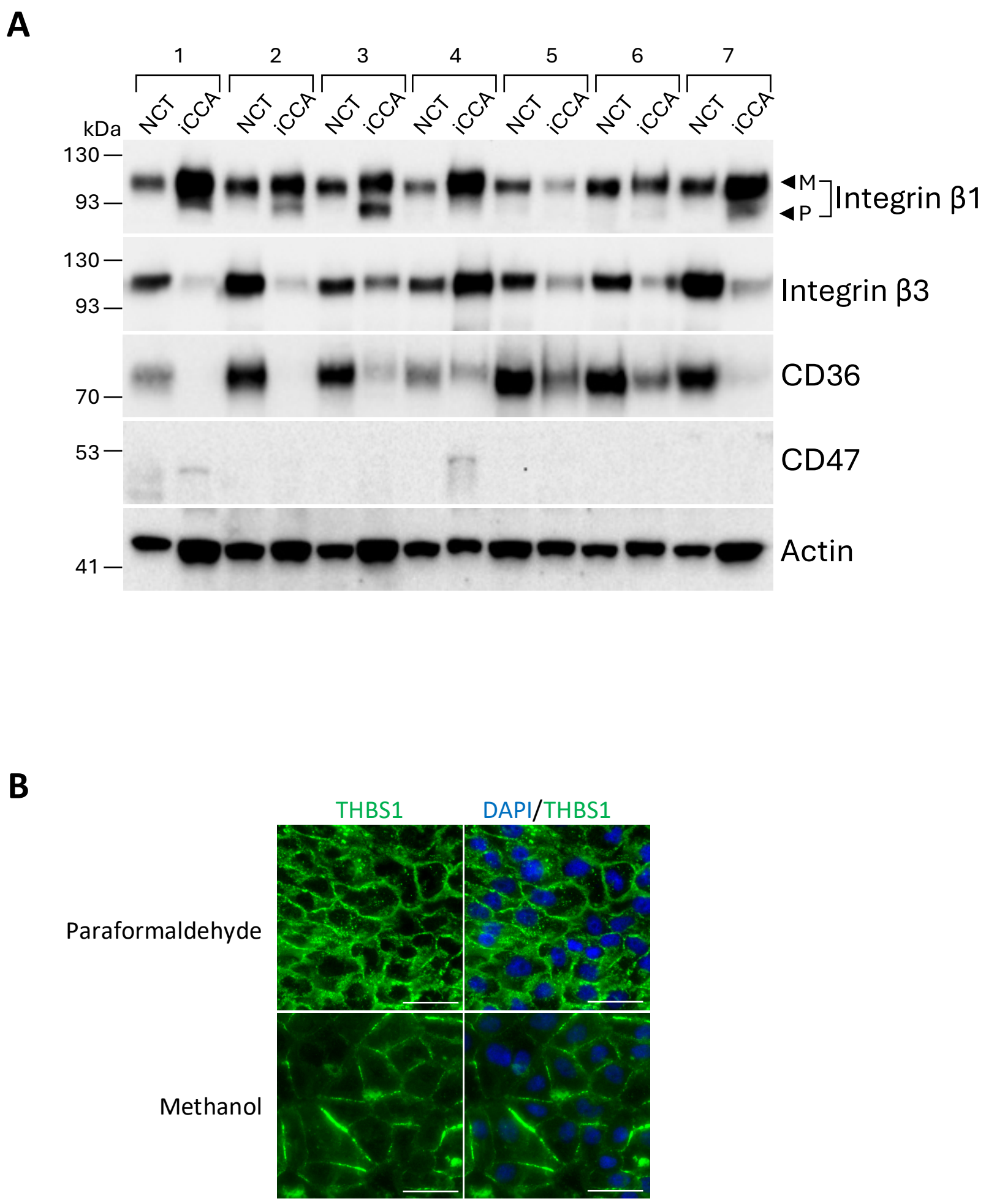

Fig. S1. Expression of integrin β1, integrin β3, CD36, and CD47 in human iCCA tissue. (A) Immunoblot analysis of integrin β1, integrin β3, CD36, and CD47 expression in iCCA and NCT total tissue extracts from iCCA patients. Actin was used as a loading control. Arrows indicate the mature (M) and precursor (P) forms of integrin β1. (B) IF detection of THBS1 in HuCCT-1 cells fixed with either 4% paraformaldehyde or methanol. Scale bars, 50 µm.

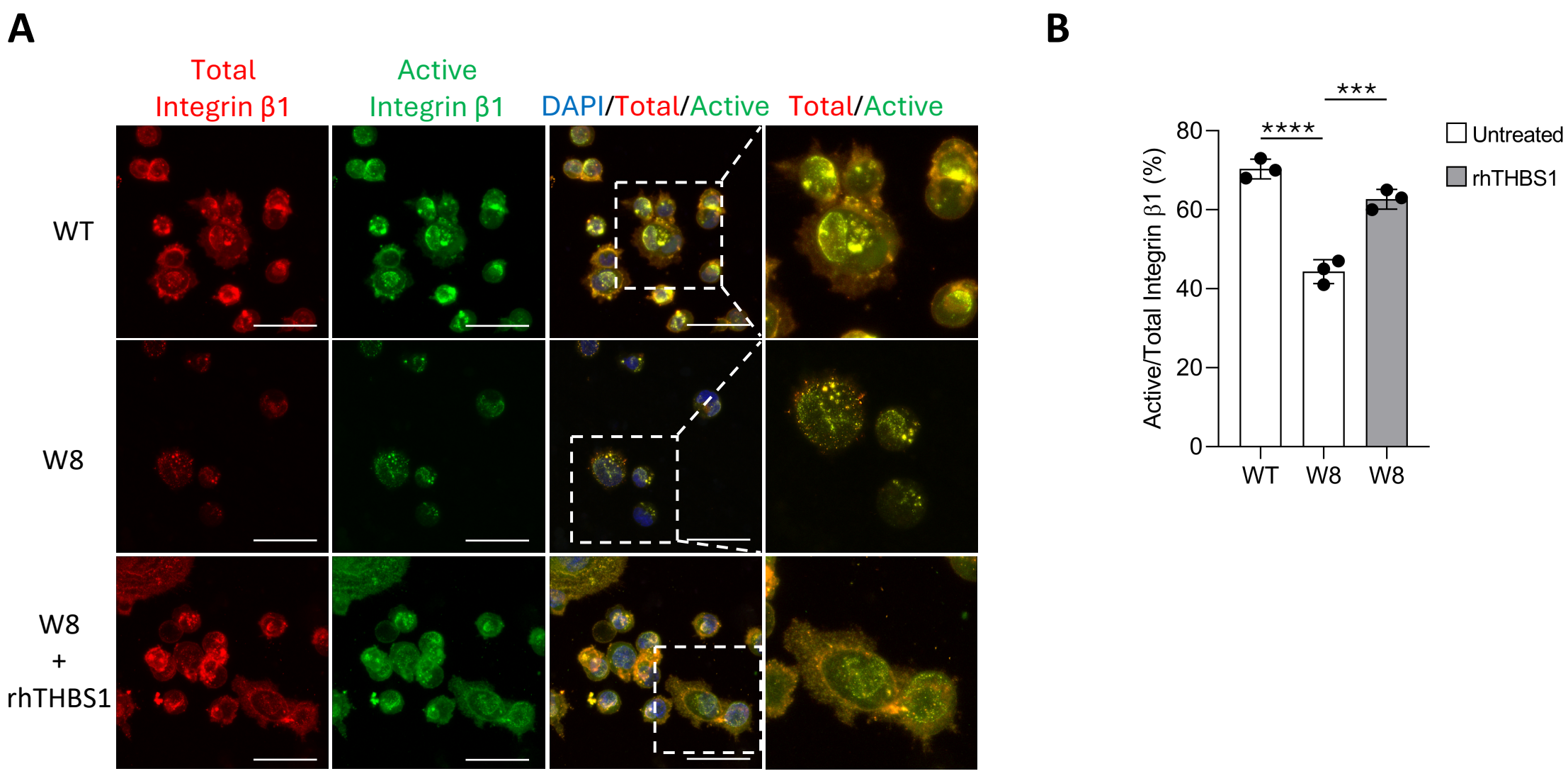

Fig. S2. Exogenous THBS1 restores active integrin β1 in W8 cells. (A) Confocal analysis of integrin β1 activation in a cell adhesion assay of W8 cells in response to 1000 ng/mL of rhTHBS1. WT cells were used as a control of integrin β1 activation; insets highlight differences in integrin β1 activation. (B) Quantitative analysis of active integrin β1 levels relative to total integrin β1 signal. Data represent mean ± SD from three independent experiments. Statistical significance was determined by one-way ANOVA with Tukey's post hoc test; ***$p < 0.001$; ****$p < 0.0001$.

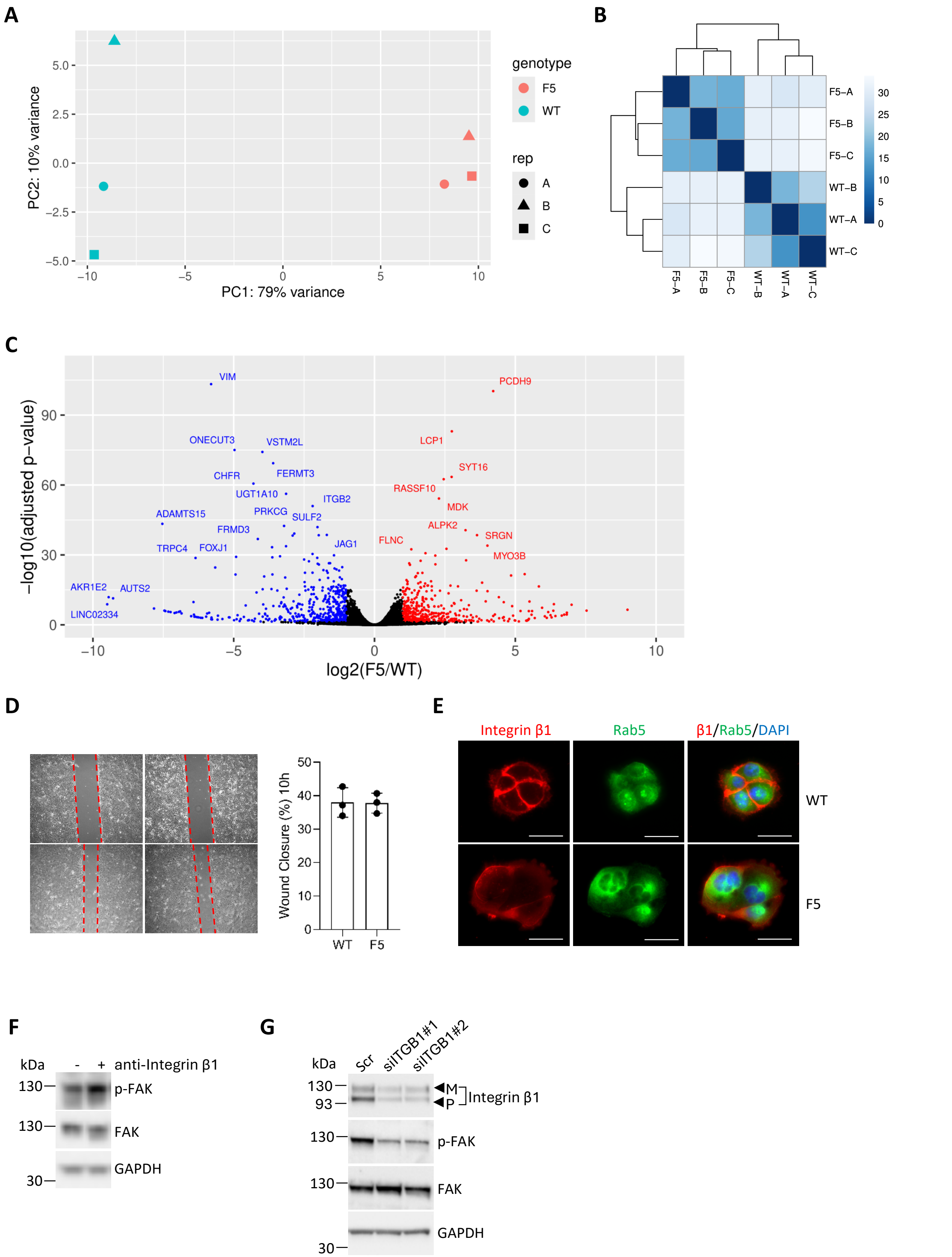
A
B
C
D
E
F
G
PC1: 79% variance
PC2: 10% variance
genotype
F5
WT
rep
A
B
C
F5–A
F5–B
F5–C
WT–B
WT–A
WT–C
−log10(adjusted p−value)
log2(F5/WT)
VIM
PCDH9
ONECUT3
VSTM2L
LCP1
CHFR
FERMT3
SYT16
UGT1A10
ITGB2
RASSF10
ADAMTS15
PRKCG
SULF2
MDK
FRMD3
ALPK2
SRGN
JAG1
FLNC
TRPC4
FOXJ1
MYO3B
AKR1E2
AUTS2
LINC02334
Wound Closure (%) 10h
WT
F5
Integrin β1
Rab5
β1/Rab5/DAPI
WT
F5
kDa
-
+
anti-Integrin β1
p-FAK
FAK
GAPDH
Scr
siITGB1#1
siITGB1#2
M
P
Integrin β1
p-FAK
FAK
GAPDH

Fig. S3. THBS1 loss alters cytoskeletal organization, cell adhesion and downstream signaling.

(A) Principal component analysis (PCA) of RNA-seq data showing variance of WT and F5 cells captured by PC1 (79%) and PC2 (10%). (B) Sample-to-sample correlation matrix based on RNA-seq expression profiles. (C) Volcano plot of 3060 DEGs in F5 versus WT cells (adjusted $p<0.05$). The x-axis shows log2 fold change (F5/WT) and the y-axis −log10 adjusted *p* values; upregulated genes are in red and downregulated genes in blue. (D) Wound healing assay of WT and F5 cells. Bar graph shows the percentage of wound closure at the indicated times. (E) Representative co-immunostained images of integrin β1 and Rab5 in WT and F5 cells. Scale bars, 50 µm. (F) Representative immunoblot analysis of total and phosphorylated FAK in HuCCT-1 cells treated with and without anti-integrin β1 blocking antibody (1000 ng/mL) 24 hours after cell seeding. GAPDH was used as a loading control. (G) Immunoblot analysis of integrin β1, total and phosphorylated FAK in HuCCT-1 cells transiently transfected with scramble siRNA oligo (Scr) or two independent siRNAs targeting ITGB1 oligos (siITGB1#1 and siITGB1#2). GAPDH was used as a loading control.

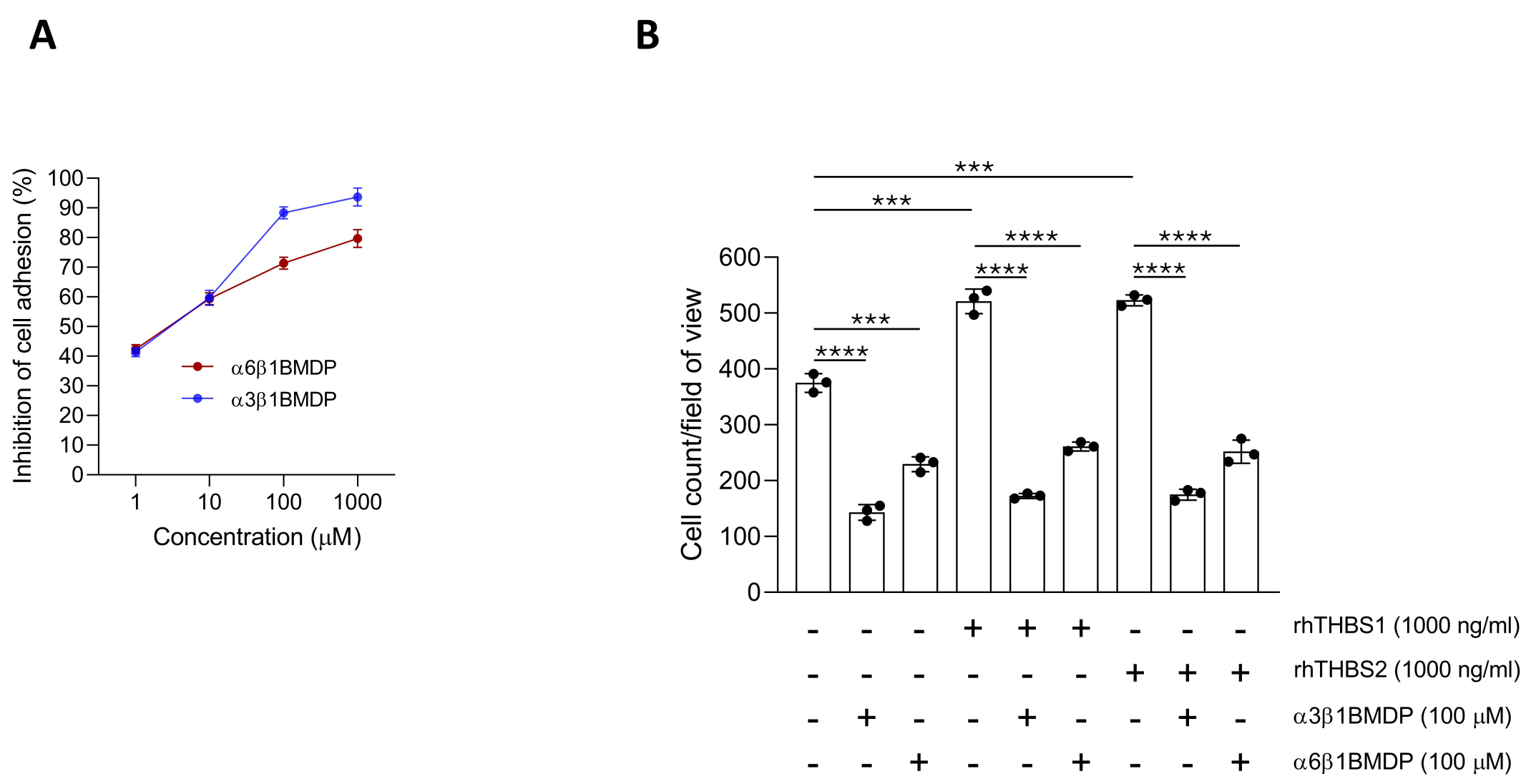

Fig. S4. α3β1 and α6β1 BMDPs inhibit CCLP1 cell adhesion. (A) Percentage of cell adhesion inhibition in CCLP1 cells following treatments with α3β1 or α6β1 BMDP at the indicated concentrations; each point represents a biological triplicate. (B) Cell adhesion assay of CCLP1 cells exposed to 1000 ng/mL rhTHBS1 or rhTHBS2, alone or with 100 μM of α3β1 or α6β1 BMDPs. Tukey's post hoc test for multiple comparisons ***$p < 0.001$; ****$p < 0.0001$.

Table.S1. List of differentially expressed genes (n = 3060).

The complete table is provided as an Excel file.

Table.S2. Enriched GO biological process (BP) categories.

Gene Set	Description	Size	Expect	Ratio	*p* Value	FDR
GO:0010975	regulation of neuron projection development	450	69,201	1,6618	9,6185E-09	6,0981E-06
GO:0031589	cell-substrate adhesion	350	53,823	1,6722	2,9067E-07	9,2141E-05
GO:0001667	ameboidal-type cell migration	428	65,817	1,5801	6,9752E-07	0,00014741
GO:0007264	small GTPase mediated signal transduction	493	75,813	1,5169	1,6255E-06	0,00025764
GO:0150115	cell-substrate junction organization	103	15,839	2,2097	2,1619E-06	0,00027413
GO:0034329	cell junction assembly	445	68,432	1,5052	8,0654E-06	0,00085225
GO:0009100	glycoprotein metabolic process	367	56,437	1,5415	1,5501E-05	0,001404
GO:0060485	mesenchyme development	312	47,979	1,584	0,00001952	0,001547
GO:0007015	actin filament organization	453	69,662	1,4642	3,0056E-05	0,0021173
GO:0043087	regulation of GTPase activity	182	27,988	1,7508	4,0292E-05	0,0024137
GO:0031345	negative regulation of cell projection organization	193	29,679	1,7184	4,8471E-05	0,0024137
GO:0022604	regulation of cell morphogenesis	243	37,368	1,6324	0,0000494	0,0024137
GO:0031032	actomyosin structure organization	203	31,217	1,6978	4,9493E-05	0,0024137
GO:0016197	endosomal transport	288	44,288	1,558	8,0272E-05	0,0036352
GO:0007492	endoderm development	85	13,071	2,0656	0,00011443	0,0048367
GO:1903829	positive regulation of protein localization	475	73,045	1,3827	0,00032366	0,012825
GO:0090130	tissue migration	313	48,133	1,4751	0,00036866	0,013749
GO:0050803	regulation of synapse structure or activity	245	37,676	1,5394	0,00039727	0,013868
GO:0001763	morphogenesis of a branching structure	204	31,371	1,5938	0,00041561	0,013868
GO:0010563	negative regulation of phosphorus metabolic process	344	52,9	1,4367	0,00053983	0,017027
GO:0002009	morphogenesis of an epithelium	499	76,736	1,3553	0,00056398	0,017027

GO:0051960	regulation of nervous system development	457	70,277	1,366	0,00068887	0,019852
GO:0060560	developmental growth involved in morphogenesis	241	37,061	1,511	0,00080089	0,022077
GO:0007163	establishment or maintenance of cell polarity	226	34,754	1,525	0,00087083	0,022349
GO:0098876	vesicle-mediated transport to the plasma membrane	149	22,913	1,6584	0,00088127	0,022349
GO:0007178	transmembrane receptor protein serine/threonine kinase signaling pathway	362	55,668	1,4012	0,00099471	0,023556
GO:0007272	ensheathment of neurons	155	23,836	1,6362	0,0010032	0,023556
GO:0031346	positive regulation of cell projection organization	352	54,13	1,404	0,0010796	0,024342
GO:0016049	cell growth	486	74,737	1,338	0,0011137	0,024342
GO:0072523	purine-containing compound catabolic process	151	23,221	1,6365	0,0011518	0,024342
GO:0060541	respiratory system development	219	33,678	1,5144	0,0012696	0,02474
GO:1990778	protein localization to cell periphery	354	54,438	1,3961	0,001274	0,02474
GO:0046434	organophosphate catabolic process	235	36,138	1,4943	0,0012877	0,02474
GO:0050808	synapse organization	478	73,506	1,3332	0,0014003	0,025656
GO:0048732	gland development	450	69,201	1,3439	0,0014164	0,025656
GO:0007229	integrin-mediated signaling pathway	113	17,377	1,7264	0,0014946	0,026227
GO:0009132	nucleoside diphosphate metabolic process	128	19,684	1,6765	0,0015306	0,026227
GO:0007409	axonogenesis	452	69,508	1,338	0,0016325	0,027237
GO:0030100	regulation of endocytosis	281	43,212	1,4348	0,0017262	0,028061
GO:0032970	regulation of actin filament-based process	365	56,129	1,3718	0,0019697	0,03122
GO:0006022	aminoglycan metabolic process	121	18,607	1,666	0,002323	0,035921
GO:0052547	regulation of peptidase activity	236	36,292	1,4604	0,0024102	0,036383
GO:0010586	miRNA metabolic process	102	15,685	1,7213	0,0026245	0,038163
GO:0006790	sulfur compound metabolic process	308	47,364	1,3935	0,0026822	0,038163
GO:1902903	regulation of supramolecular fiber organization	375	57,667	1,3526	0,0027508	0,038163

GO:0071559	response to transforming growth factor beta	270	41,52	1,421	0,0027689	0,038163
GO:0000041	transition metal ion transport	98	15,07	1,7252	0,0030132	0,040647
GO:0030865	cortical cytoskeleton organization	51	7,8427	2,0401	0,0031175	0,040736
GO:0097485	neuron projection guidance	239	36,753	1,4421	0,0031945	0,040736
GO:0042692	muscle cell differentiation	394	60,589	1,3369	0,0032127	0,040736
GO:0007422	peripheral nervous system development	89	13,686	1,7536	0,0033998	0,041522
GO:0006898	receptor-mediated endocytosis	256	39,367	1,4225	0,0034056	0,041522

Table.S3. Enriched GO BP categories after redundancy removal using "Weighted set cover" algorithm.

Gene Set	Description	Size	Expect	Ratio	P Value	FDR
GO:0010975	regulation of neuron projection development	450	69,201	1,6618	9,6E-09	8,07E-06
GO:0031589	cell-substrate adhesion	350	53,823	1,6722	2,9E-07	0,000122
GO:0001667	ameboidal-type cell migration	428	65,817	1,5801	7E-07	0,000195
GO:0007264	small GTPase mediated signal transduction	493	75,813	1,5169	1,6E-06	0,000341
GO:0034329	cell junction assembly	445	68,432	1,5052	8,1E-06	0,001128
GO:0009100	glycoprotein metabolic process	367	56,437	1,5415	1,6E-05	0,001858
GO:0060485	mesenchyme development	312	47,979	1,584	2E-05	0,002047
GO:0007015	actin filament organization	453	69,662	1,4642	3E-05	0,002802
GO:0016197	endosomal transport	288	44,288	1,558	8E-05	0,004811
GO:1903829	positive regulation of protein localization	475	73,045	1,3827	0,00032	0,016972
GO:0010563	negative regulation of phosphorus metabolic process	344	52,9	1,4367	0,00054	0,022532
GO:0002009	morphogenesis of an epithelium	499	76,736	1,3553	0,00056	0,022532
GO:0051960	regulation of nervous system development	457	70,277	1,366	0,00069	0,026271
GO:0007178	transmembrane receptor protein serine/threonine kinase signaling pathway	362	55,668	1,4012	0,00099	0,030059
GO:0016049	cell growth	486	74,737	1,338	0,00111	0,031146
GO:0046434	organophosphate catabolic process	235	36,138	1,4943	0,00129	0,031777
GO:0048732	gland development	450	69,201	1,3439	0,00142	0,033009
GO:0030100	regulation of endocytosis	281	43,212	1,4348	0,00173	0,036206
GO:0052547	regulation of peptidase activity	236	36,292	1,4604	0,00241	0,045959
GO:0006790	sulfur compound metabolic process	308	47,364	1,3935	0,00268	0,048399

Table.S4. Antibodies used for Western Blot and IF analysis

Antibody	Source (catalog no.)	Concentration	Application
Anti-Actin	Santa Cruz Biotechnology, sc-1615	1:1000	WB
Anti-GAPDH	Santa Cruz Biotechnology, sc-20357	1:1000	WB
Anti-Vimentin	Cell Signaling, 5741	1:1000	WB
Anti-integrin β1 (D2E5)	Cell Signaling, 9699	1:1000	WB
Anti-integrin β1 (9EG7)	BD Pharmingen, 550531	1:50	IF
Anti-integrin β1 (12G10)	Abcam, ab30394	1:100	IF
Anti-integrin β1 (Mab13)	BD Pharmingen, 552828	1:50	IF
Anti-integrin α3	Proteintech, 66070-1-Ig	1:1000; 1:100	WB, IF
Anti-integrin α6	Abcam, ab124924	1:500	WB
Anti-integrin α6	Santa Cruz Biotechnology, sc-19622	1:100	IF
Anti-CD47	Cell Signaling, 63000	1:500	WB
Anti-THBS1	Santa Cruz Biotechnology, sc-81755	1:200; 1:50	WB, IF
Anti-COL1A1	Santa Cruz Biotechnology, sc-8784	1:50	WB
Anti-CD36	Abcam, ab252922	1:1000	WB
Anti-pFAK	Cell Signaling, 8556	1:500	WB
Anti-FAK	Cell Signaling,13009	1:1000	WB
Anti-Rab5	Cell Signaling, 3547	1:100	IF
Anti-Claudin1	Thermo Fisher Scientific,71-7800	1:100	IF
Anti-E-cadherin	BD Pharmingen, 610181	1:200	WB
Anti-MMP9	Cell Signaling, 13667	1:500	WB

Table.S5. Primer sequences used for real-time qPCR

Gene	Forward	Reverse
THBS1	TGTGATCCTGGACTCGCTGT	TAAAAGCGGCTGCTGGACTG
THBS2	CGTGGACAATGACCTTGTTG	GCCATCGTTGTCATCATCAG
ITGA3	CCCGGTGCCTACAACTGGAA	TGCCTACCTGCATCGTGTACC
ITGA6	TGAAAGTCTCGTTCCTGTTCCTG	GCTCCACTGTGATTGGCTCT
ITGA8	TGTCAGGCGTTCAACCTGGA	CAAGACACTCGCTGTGCGG
ITGA9	GGGCCCCCATGTTTTCTGAG	TAGGCACCATCCCCAGTCAG
ITGA10	ATGGAACCCAGAGTGGAGTCA	CGACCATCCACACTTCGGC
ITGA11	CTTCCGTGAGGAGGATCCCA	CAGGGTGCTGCCTACAATGA
GAPDH	TGCACCACCAACTGCTTAGC	GGCATGGACTGTGGTCATGAG
